# Exploring the effects of moxibustion on cognitive function in rats with multiple cerebral infarctions from the perspective of glial vascular unit repairing

**DOI:** 10.3389/fphar.2024.1428907

**Published:** 2024-10-22

**Authors:** Jingji Wang, Kunrui Du, Chang Liu, Xiaoyu Chen, Wenming Ban, Guoqi Zhu, Jun Yang

**Affiliations:** ^1^ Center for Xin’an Medicine and Modernization of Traditional Chinese Medicine of IHM, and Key Laboratory of Molecular Biology (Brain diseases), Anhui University of Chinese Medicine, Hefei, China; ^2^ Acupuncture and Moxibustion Clinical Medical Research Center of Anhui Province, The Second Affiliation Hospital of Anhui University of Chinese Medicine, Hefei, China; ^3^ Department of Neurology, Taihe County Hospital of Traditional Chinese Medicine, Fuyang, China; ^4^ The First Affiliation Hospital of Anhui University of Chinese Medicine, Hefei, China

**Keywords:** moxibustion, vascular dementia, glial vascular unit, astrocyte, glial fibrillary acidic protein, CX43

## Abstract

**Objective:**

This study aimed to explore the effect of moxibustion at Governor Vessel (GV) acupoints, including Baihui (GV 20), Shenting (GV 24) and Dazhui (GV 14) for 14 days on glial vascular unit (GVU) in rats with multiple microinfarctions (MMI), and to explore its action mechanism.

**Methods:**

The effect and mechanism of moxibustion on vascular dementia (VD) were studied in MMI rats by means of behavioral and molecular biology experiments.

**Results:**

Rats receiving MMI showed impairment of memory function, reduction of cerebral blood flow, damage of blood-brain barrier (BBB) integrity and increased brain mass. MMI also increased neuronal degeneration in the hippocampus. Notably, levels of glial fibrillary acidic protein (GFAP) and complement component 3 significantly increased, but those of Connexin43 (CX43) and platelet derived growth factor receptor β (PDGFRβ) significantly decreased in the hippocampus of the rats receiving MMI. Moxibustion, as well as oxiracetam (ORC) treatment improved memory function and neuronal degeneration, ameliorated BBB integrity, increased cerebral blood flow and decreased brain mass. In addition, moxibustion as well as oxiracetam (ORC) treatment reduced the decrease of CX43 protein and increased PDGFRβ protein level in the hippocampus of MMI rats. Moreover, moxibustion treatment reversed MMI-induced increase of the GFAP/CX43 ratio in vascular structural units. Importantly, after PDGFRβ inhibition, VD rats treated with moxibustion had impaired learning and memory, decreased cerebral blood flow, and BBB disruption.

**Conclusion:**

Moxibustion treatment at various GV acupoints improved cerebral blood flow and repaired BBB function in rats with MMI, likely through protecting GVU.

## 1 Introduction

Vascular dementia (VD) is a chronic cerebral hypoperfusion vascular disease caused by cerebrovascular abnormalities (Rajeev et al., 2023). Its pathogenesis is related to changes in vascular permeability, dysfunction of neurovascular coupling, neuronal damage, abnormal activation of glial cells, and neuroinflammation (Bai and Zhang, 2021; Du et al., 2022). Vascular lesion is increasingly becoming a hot topic in dementia research (Inoue et al., 2023), as it can lead to structural and functional obstacles to the glial vascular unit (GVU), a structural and functional unit established by neurons, glia, endothelial cells, pericytes, vascular smooth muscle cells and tight junction (Li et al., 2021). Furthermore, the impairment of GVU exacerbates the progression of VD and leads to cognitive impairment in VD (Yang et al., 2022). At the same time, GVU also plays a vital role in maintaining the function of the central nervous system (CNS), the integrity of the blood-brain barrier (BBB) and brain protection (Kugler et al., 2021; Chen et al., 2023). Therefore, repairing the structure and function of GVU and improving BBB permeability are essential for the functional recovery of VD patients.

Moxibustion is one of the important treatment methods of Traditional Chinese Medicine (TCM), which has been proven to safely and effectively improve cognitive impairment and other symptoms of VD by improving cerebral blood flow, protecting neurons, reducing oxidative stress and inflammation (Li et al., 2022; Ma et al., 2020; Su et al., 2021). Previously, we also found that moxibustion at the acupoints of the Governor Vessel could alleviate amyloid β protein-related neurotoxic damage and improve cognitive impairment in VD rats (Wang et al., 2020). However, it is still unclear whether moxibustion at the acupoints of the Governor Vessel can improve cognitive impairment in VD by affecting GVU.

By closely connecting brain cells and blood vessels, and regulating brain injury, astrocytes play an irreplaceable role in neuroprotection (Yao et al., 2023). Connexin 43 (CX43) regulates the function of astrocytes, and changes in CX43 levels affect GVU (Zhao et al., 2018). Platelet derived growth factor receptor β (PDGFRβ), a marker of pericytes, and Zonula occluden-1 (ZO-1), a tight junction protein between endothelial cells, are also important components of GVU and BBB (Cicognola et al., 2023; Wu et al., 2023). Brain damage caused by multiple microinfarct (MMI) leads to a decrease in memory, which is similar to what is observed in VD patients (Hartmann et al., 2018). To better investigate the pathogenesis of VD patients in clinical practice, a rat model of MMI has been developed (Venkat et al., 2017). In this study, we aimed to investigate the effects of moxibustion at Governor Vessel (GV) acupoints on cognitive function in rats receiving MMI. Additionally, we explored its mechanism of action from the perspective of GVU and compared it with the use of PDGFRβ inhibitor to validate our hypothesis, providing a more possible theoretical basis for the treatment of VD with moxibustion in clinical practice.

## 2 Materials and methods

### 2.1 Experimental animals

SD rats were purchased from the Laboratory Animal Center of Anhui Medical University (Six-week-old male SD rats were selected and weighed 200 ± 20 g) and raised in an environment including 20°C–25°C room temperature, 40%–75% humidity, and 12 h/12 h alternating time between day and night. Two experiments were designed in this study.

All animals were decapitated under anesthesia with isoflurane (3%) after behavioral testing. Brain mass was measured, followed by pathological and biochemical tests. The whole experiment was approved by the Animal Experimentation Ethics Committee of Anhui University of Traditional Chinese Medicine (Approval number: AHUCM-rats-2023002).

### 2.2 Preparation of MMI rat model and treatments

MMI was used to model VD in rats (Venkat et al., 2017). Fresh cholesterol crystals were prepared by dissolving cholesterol (H34021875, Anhui Fengyuan Pharmaceutical Co. Ltd., China) in ethanol, heating in a 60°C water bath for 20 min, filtering with filter paper, and evaporating in an oven. The cholesterol crystals underwent grinding and the solution of cholesterol crystals was prepared in saline. Cholesterol crystals ranging from 70–100 μm in size were collected by filtrating microporous filter membrane. The number of cholesterol crystals was counted using a blood cell counter and cholesterol crystals were suspended in saline (500 ± 100 crystals/300 μL). After induction of anesthesia using 0.41 mL/min and maintenance of 2% isoflurane inhalation anesthesia (R580S, RWD Life Science Co. Ltd., China), rats were positioned supine on an operating table. Following thorough disinfection, a 2 cm median incision was made in the neck. The subcutaneous tissue was carefully separated and the right common carotid artery (CCA), right internal carotid artery (ICA), right external carotid artery (ECA), and vagus nerve were identified. The ECA was ligated, while the CCA and ICA were temporarily clamped using an arteriole clamp. A small incision was prepared at the distal end of the ECA. We slowly inserted a PE-50 tube into the ECA through the incision while pulling the distal end of the ECA outward and upward to align it parallel to the ICA. After that, we removed the micro arterial clamp and released the ICA. Then, we gently and slowly pushed the PE 50 tube in the direction of the ICA and injected cholesterol crystals (0.3 mL). For the Sham group, an equal amount of saline was injected into the ICA. Gentamicin (80 mg/2 mL, 0.3 mL) (No: 11Y12011 D1, Yichang Renfu Pharmaceutical Co. Ltd., China) was subcutaneously injected to prevent infection. Following the establishment of the model, the Morris water maze test was used to verify the model as previously described (Inoue et al., 2023). Successful model replication was confirmed if the escape latency was significantly longer than that of the Sham group (P < 0.05).

#### 2.2.1 Experiment 1

**FIGURE 1:**
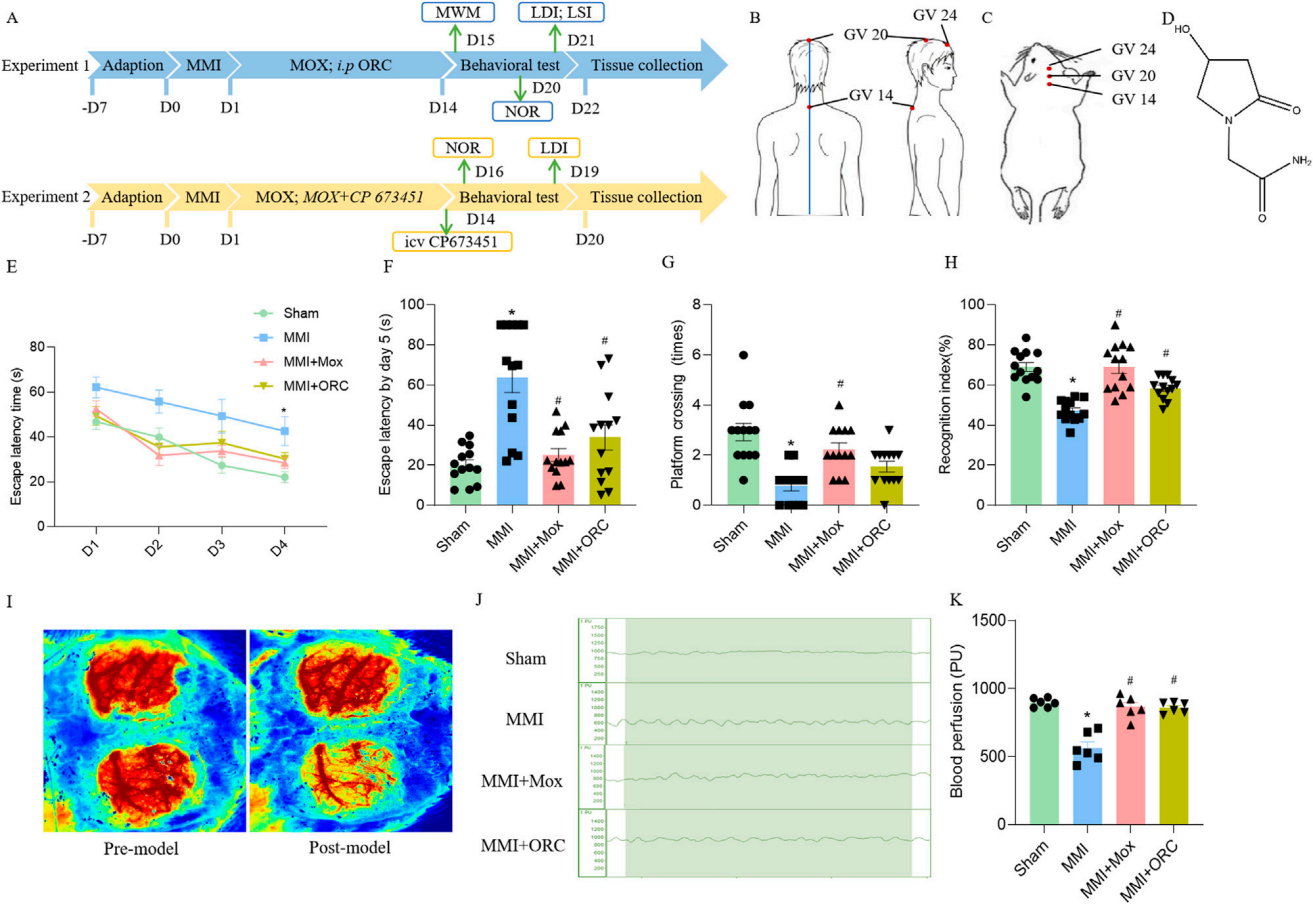
Experimental schematic, moxibustion improved learning and memory, and cerebral blood flow in VD rats. **(A)** Flow diagram of the experimental procedure. **(B)** Location of GV acupoints in human. **(C)** Location of acupoints used in rats. **(D)** ORC chemical structure. **(E)** The escape latency in positioning navigation test in the Morris water maze (n = 13) (Tukey’s test). **(F)** The first escape latency period in space exploration test in the Morris water maze (n = 13) (Dunn’s test). **(G)** Number of cross platforms in space exploration test in the Morris water maze (n = 13) (Dunn’s test). **(H)** Recognition index for novel object recognition test (n = 13) (Tukey’s test). **(I)** Laser speckle imaging observation of rats before and after MMI (n = 6). **(J, K)** Laser Doppler comparison of cerebral blood flow of rats in each group (n = 6) (Tukey’s test). Data are presented as means ± SEM. Compared with the Sham group, *P < 0.05. Compared with the MMI group, ^#^ *P < 0.05*.

MMI + MOX group: Baihui (GV 20), Shenting (GV 24) and Dazhui (GV 14) were positioned by referring to *Experimental Acupuncture* (Figures 1A–C) (Li, 2003). Rats were placed on a self-constructed wooden platform (10 cm × 5 cm × 1.5 cm), and the moxa-stick moxibustion (4 mm × 120 mm, Nanyang Wuyecao Clover Bioproducts Co. Ltd., China) were lit with a match. The moxa-stick moxibustion was suspended approximately 2 cm above the acupoints. We quickly adjusted the suspension distance as necessary to avoid movements, maintaining the skin temperature of the acupoints at 41°C ± 1°C. Moxibustion lasted 30 min per acupoint, once daily for 14 consecutive days. MMI + oxiracetam (ORC) group: ORC (Hunan Jianlang Pharmaceutical Co. Ltd., China; the structure of ORC was shown in Figure 1D) was dissolved in saline (10 mg/mL). Rats received intraperitoneal injections of 50 mg/kg ORC (dissolved in saline) once a day for 14 consecutive days (Wang et al., 2022). Sham and MMI groups: The processing time was equivalent to that of the MMI + MOX group, and animals were treated without any additional intervention except for identical grasping and standing on the platform. At the end of the treatment, the Morris water maze (MWM), the Novel object recognition (NOR), the Laser speckle imaging (LSI), the Laser Doppler imaging (LDI) were performed in order. Tissue collections were taken at the end of the behavioral tests to test the GVU-related protein expression.

#### 2.2.2 Experiment 2

MOX + CP 673451 group: The moxibustion treatment was the same as that of the MOX + MMI group in Experiment 1, and after 14 days of treatment, CP 673451 was locally injected (1 mg/kg, Selleck; China, #S1536; AP: −0.8 mm, ML: −1.5 mm, DV: −4 mm) (Asgari Taei et al., 2021; Bi et al., 2022) (Figure 1A). In the moxa group, only an equal amount of PBS was injected, and the rest of the operation was the same as that in the inhibitor group. At the end of the treatment, the NOR, the LDI were performed in order. Tissue collections were taken at the end of the behavioral tests.

### 2.3 Morris water maze

The MWM was used to assess learning and memory as previously described (Bian et al., 2022; Shen et al., 2021). The MWM was conducted on day 15 after establishment of the model. The Morris water maze test encompasses a positioning navigation experiment and a space exploration experiment. The positioning and navigation experiment lasted for 4 days, with the hidden platform fixed in the fourth quadrant, approximately 2 cm below the horizontal plane. The swimming behavior and escape latency of each group was recorded for 90 s. If the rat fully stood on the platform for at least 5 s within the time frame, it was considered a successful escape, and the time it took was recorded as the escape latency. During exploration experiment, the platform was removed. The swimming route, first escape latency, and the number of times the rats crossed the platform was recorded within 90 s (Yan et al., 2022). The data were analyzed using specialized software (Noldus Netherlands, EthoVision XT, China).

### 2.4 Novel object recognition test

The experiment consisted of three stages: habituation day, training day, and testing day. During training, the rats were allowed to explore two identical objects for 10 min in a black open field box (100 cm × 100 cm × 100 cm). On test day, one of the training objects is replaced with a novel object. Each group of rats was placed in the box to explore freely for 5 min and their activity trajectory was recorded. After each rat’s exploration, all objects and the open field boxes were cleaned with 75% ethanol to eliminate any trace of odor. Recognition index (RI) = the time spent exploring new objects/(time spent exploring new + old objects) (Shan et al., 2019). The data were analyzed using specialized software (Noldus Netherlands. EthoVision XT, China).

### 2.5 Laser speckle imaging

LSI was used to assess cerebral blood flow. Rats were anesthetized with isoflurane at a rate of 0.41 mL/min and maintained at a concentration of 2% isoflurane inhalation anesthesia. The skulls of rats were exposed, the cranial surfaces were adequately polished and photographed with laser speckle imaging (RWD Life Science Co., Ltd., RFLSI ZW, China), and then the skin was sutured and the VD model was performed. After 14 days, the same operation was performed to observe the changes in cerebral blood flow.

### 2.6 Laser Doppler imaging

LDI was also used to assess cerebral blood flow. Briefly, rats were immobilized in the supine position on the operating table after anesthesia. The top of the head was sterilized and a 2-cm incision was made along the midsagittal line. The subcutaneous tissue was separated so that the skull was completely exposed, and laser probes were fixed isometrically and vertically to the anterior fontanelle and the right side of the skull. The LDPM device (PERIMED, Sweden, PF 5001–2252) was connected to a PF 5010, and the cerebral blood flow of rats was measured and recorded within 2 min. Data were analyzed using the PSW software for the laser Doppler flow detector.

### 2.7 HE staining

Changes in rat hippocampal neurons in each group were assesses by HE staining. Brain tissue was embedded in paraffin and dehydrated, then cryosectioned at 6 μm thickness (LEICA CM 1950, Germany). HE staining was carried out for 3–5 min, then washed with water, differentiated with differentiation solution, and then washed with tap water. The membrane was then covered and images were observed and collected under a microscope (OLYMPUS BX53, Japan). The degeneration rate of hippocampal neurons was analyzed using ImageJ software.

### 2.8 Evans blue staining

Rats were anesthetized with isoflurane at a rate of 0.41 mL/min and maintained at a concentration of 2% isoflurane. 2% Evans blue dye (Chengdu Zhengneng Biotechnology Co., Ltd. #E6135-1g, 4 mL/kg) was injected into the femoral vein and circulated for 1 h, followed by cardiac perfusion with PBS. After the clear fluid flowed out of the right heart, brains were collected, dehydrated, fixed, and sectioned to a thickness of 20 μm. Sections were rinsed three times with PBS for 15 min each, then incubated with DAPI for 30 min in the dark and further rinsed three times with PBS for 15 min each. Sections were then covered with coverslips and observed under a microscope (LEICA DMi8 Automation, Germany). The total fluorescence values of Evans blue and DAPI were analyzed using Image Pro Plus. The BBB permeability was calculated using the ratio of Evans blue fluorescence and DAPI fluorescence.

### 2.9 Western blot

Western blot analysis was performed to quantify levels of GFAP, CX43, PDGFRβ, ZO-1, C3, S100A10, Claudin-5 and Occludin in the hippocampi. Briefly, rat hippocampal tissues from each group were weighed, added to cell lysate and grounded on ice for 30 min. The mixture was centrifuged at 12,000 r/min for 15 min at 4°C and the supernatant was collected. Finally, an equal amount of loading buffer was added and heated in a 100°C water bath for 10 min to completely denature the proteins. After electrophoresis and transfer using SDS-PAGE (EPS 600, Shanghai Danone Life Science and Technology Co., Ltd., China), membranes were blocked with 5% skim milk for 2 h at room temperature. Membranes were incubated with primary antibodies overnight at 4°C: GFAP (1:1,000; #AG259, Beyotime Biotechnology, China), CX43 (1:1,000; #340279, Chengdu Zhengneng Biotechnology, China), PDGFRβ (1:1,000; #3169, CST, United States), ZO-1 (1:1,000; #WL03419, Shenyang Wanlei, China), C3 (1:1,000; #21337-1-AP, Proteintech Group, China), S100A10 (1:1,000; #11250-1-AP, Proteintech Group, China), Claudin-5 (1:1,000; #343214, Chengdu Zhengneng Biotechnology, China) and Occludin (1:1,000; #E6B4R, CST, United States). Thereafter, membranes were incubated with the secondary antibody (1:10,000, Chengdu Zhengneng Biotechnology, China) at room temperature for 2 h, followed by washing with PBST. In combination with chemiluminescent agents, membranes were developed, exposed and analyzed for gray values using ImageJ software.

### 2.10 Immunofluorescence

Immunofluorescence analysis was performed to observe changes in the expression of GFAP and CX43 in the CA1 subregion of the hippocampus. Fixed frozen brain tissues were sectioned at a thickness of 20 μm. Immunofluorescence was conducted using GFAP antibody (1:100, AG259, Beyotime Biotechnology, China) and CX43 antibody (1:200, 340,279, Chengdu Zhengneng Biotechnology, China) (Xie et al., 2023; Li et al., 2023). FITC-labeled anti-rabbit IgG (1:100, ZB-0311, ZS-BIO, China) and Alexa Fluor 594-labeled anti-mouse IgG (1:100, ZF-0513, ZS-BIO, China) were added and incubated in the dark for 1 hour. DAPI was added for 30 min, then rinsed with PBS (3 times, 15 min each). The slides were covered, observed and photographed (LEICA DMi8 Automation, Germany). The average fluorescence intensities of GFAP, CX43, and co-localization ratio were analyzed using Image Pro Plus.

### 2.11 Statistical analysis

The data were analyzed using GraphPad Prism 9.0 software (GraphPad Inc., San Diego, CA, United States). Continuous variables were expressed as Mean ± SEM. All data were first subjected to normality tests by Shapiro–Wilk test. For normally distributed data, independent sample *t*-test were used for two groups, and one-way ANOVA, two-way ANOVA, or repeated measures ANOVA followed by Tukey’s *post hoc* test for three or more groups. Non-normal distributions were analyzed using Kruskal-Wallis followed by Dunn’s comparisons test. The data from the Sham group in the Western blot were normalized using the rank sum test. The value of *P* < 0.05 was considered a significant difference.

## 3 Results

### 3.1 Effect of moxibustion on learning and memory in rats with MMI

The spatial memory was firstly tested in the rats of each group. In the 1–4 days positioning navigation experiment, the time to search for the platform was significantly shortened in each group. However, the time to search for the platform was significantly prolonged in the MMI group, as compared to the Sham group on day 4 (F (3, 49) = 8.081, *P = 0.8023*) (Figure 1E). On the fifth day of the space exploration experiment, the first escape latency of rats in the MMI group (H = 19.04, *P = 0.0001*) increased significantly, compared with the Sham group. Compared with the MMI group, the first escape latency of the rats in MMI + MOX group (H = 19.04, *P = 0.0029*) and MMI + ORC group (H = 19.04, *P = 0.0278*) were significantly reduced (Figure 1F). The number of cross platform quadrant of the MMI group (H = 23.75, *P < 0.0001*) was significantly reduced compared with the Sham group. Compared with the MMI group, the number of cross platform in the rats of MMI + MOX (H = 23.75, *P = 0.0019*) group significantly increased (Figure 1G).

Then, we compared object recognition memory in the rats of each group. Compared with the Sham group, the cognitive index of the MMI group rats decreased significantly (F (3, 48) = 21.73, *P < 0.0001*). Compared with the MMI group, the cognitive index in the MMI + MOX rats (F (3, 48) = 21.73, *P < 0.0001*), as well as in the MMI + ORC group (F (3, 48) = 21.73, *P = 0.0048*) was significantly increased (Figure 1H).

### 3.2 Effect of moxibustion on the cerebral blood flow in rats with MMI

Initially, we confirmed the effect of MMI on cerebral blood flow using laser speckle imaging. We delivered cholesterol crystals unilaterally and the contralateral brain was set as a control. Compared with the sham side, cerebral blood flow of the side receiving MMI was obviously reduced (Figure 1I). Next, we applied laser Doppler imaging to quantitatively analyze cerebral blood flow. Compared with the Sham group, cerebral blood flow in the MMI group fluctuated over a wider range. Compared with the MMI group, fluctuation of cerebral blood flow in the MMI + MOX group was reduced (Figure 1J). Compared with the Sham group, cerebral blood flow in the MMI group (F (3, 20) = 27.65, *P < 0.0001*) was significantly reduced. Compared with the MMI group, the cerebral blood flow of rats in the MMI + MOX group (F (3, 20) = 27.65, *P < 0.0001*) and the MMI + ORC group (F (3, 20) = 27.65, *P < 0.0001*) was significantly increased (Figure 1K).

### 3.3 Effect of moxibustion on hippocampal neurons in rats with MMI

**FIGURE 2:**
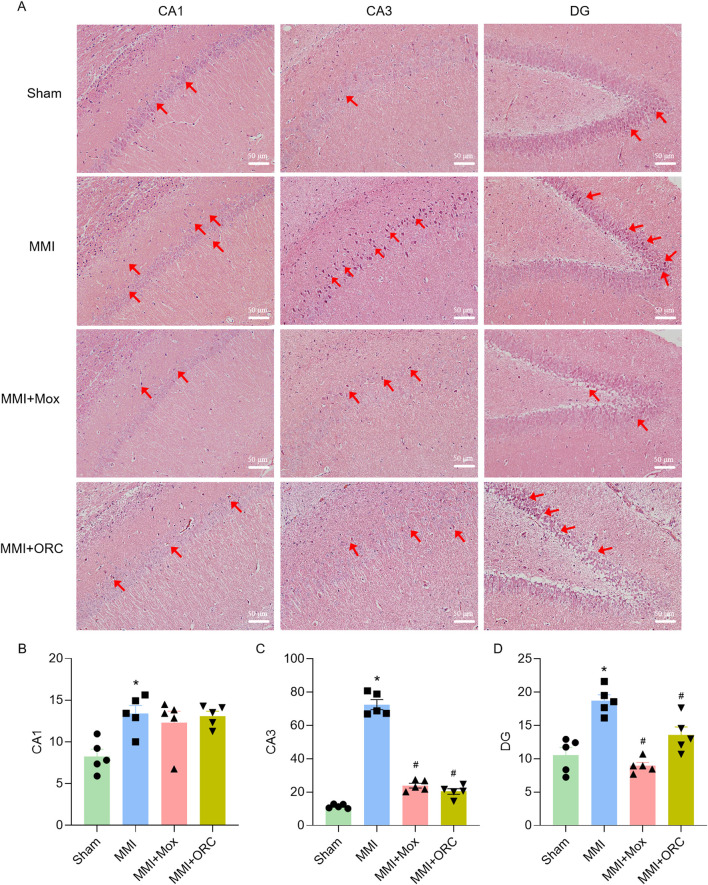
Moxibustion alleviated neuronal degeneration in the hippocampus of VD rats. **(A–D)** Neuronal changes in CA1, CA3, and DG region. Scale bar: 50 µm. (n = 3) (Tukey’s test or Dunn’s test). Data are presented as means ± SEM. Red arrows indicated NPCs. Compared with the Sham group, *P < 0.05. Compared with the MMI group, ^#^ *P < 0.05*.

Pathological changes of hippocampal cells were determined using HE staining. Hippocampal neurons of the Sham group were regularly arranged. By contrast, nuclear pyknotic cells (NPCs) were observed in the hippocampus of the MMI group (Figure 2A). We further quantified NPCs in different regions of the hippocampus (CA1, CA3, DG). Compared with the Sham group, the numbers of NPCs in CA1 (*P < 0.05*, H = 8.403, *P = 0.0285*), CA3 (F (3, 16) = 213.7, *P < 0.0001*), and DG (F (3, 16) = 19.21, *P = 0.0001*) regions of the MMI group significantly increased. Compared with the MMI group, the numbers of NPCs in the CA3 of the MMI + MOX (F (3, 16) = 213.7, *P < 0.0001*) and MMI + ORC (F (3, 16) = 213.7, *P< 0.0001*) groups. and the numbers of NPCs in the DG of the MMI + MOX (F (3, 16) = 19.21, *P < 0.0001*) and MMI + ORC (F (3, 16) = 19.21, *P = 0.0096*) groups were significantly reduced (*P < 0.05*) (Figures 2B–D).

### 3.4 Effects of moxibustion on BBB permeability in rats with MMI

**FIGURE 3:**
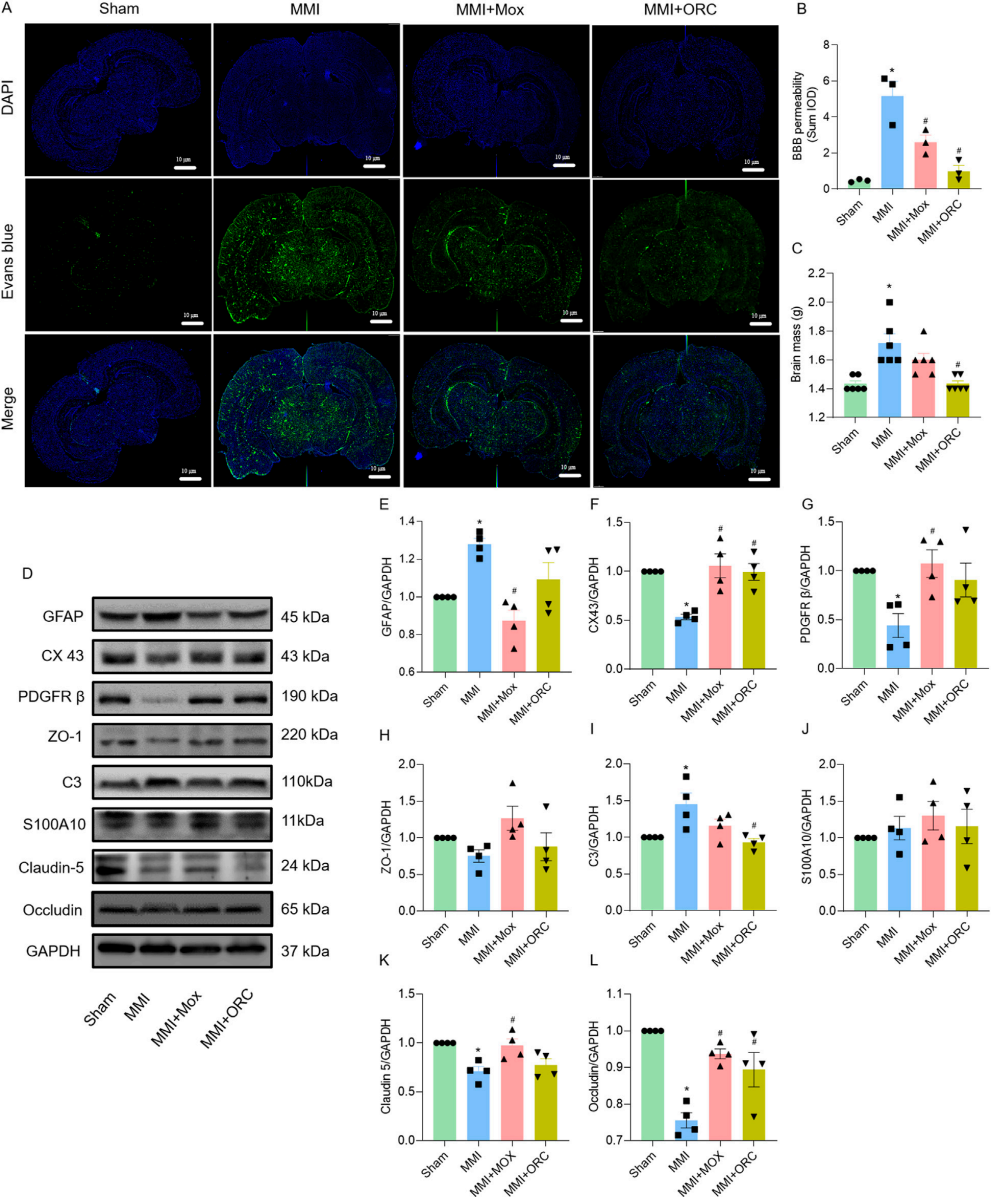
Moxibustion reduced blood-brain barrier permeability in VD rats, and effects of moxibustion on GVU-related protein expression. **(A–B)** Evans blue staining for assessing blood-brain barrier permeability (n = 3) (Tukey’s test). **(C)** Comparison of brain mass of rats in each group (n = 6) (Dunn’s test). **(D–L)** Western blots for GFAP, CX43, PDGFRβ, ZO-1, C3, S100A10, Claudin-5 and Occludin proteins in the hippocampus (n = 4) (Tukey’s test). Data are presented as means ± SEM. Compared with the Sham group, *P < 0.05. Compared with the MMI group, ^#^ *P < 0.05*.

BBB permeability was analyzed using Evans blue staining. Compared to the Sham group, BBB permeability in the MMI group increased significantly as evidenced by the increase of fluorescence (F (3, 8) = 19.33, *P = 0.0006*). Compared with the MMI group, BBB permeability of rats in the MMI + MOX (F (3, 8) = 19.33, *P = 0.0226*) and MMI + ORC (F (3, 8) = 19.33, *P = 0.0012*) groups was significantly ameliorated, as shown by the decrease in Evans blue fluorescence (Figures 3A, B).

Compared with the Sham group, brain weights of the rats in the MMI group were significantly higher (H = 17.06, *P = 0.0025*). By contrast, brain weight of rats in the MMI + MOX were not significantly altered by MOX (H = 17.06, *P > 0.9999*). The brain weights of rats in the MMI + ORC group (H = 17.06, *P = 0.0025*) was significantly decreased compared to the MMI group (Figure 3C).

### 3.5 Effect of moxibustion on the levels of GVU-related proteins in hippocampus

As GVU could be repaired by moxibustion, we also determined GVU-related protein expression. Compared with the Sham group, rats in MMI group showed increased expression of GFAP (F (3, 12) = 9.541, *P = 0.0174*), C3 (F (3, 12) = 6.011, *P = 0.0240*) and decreased expression of CX43 (F (3, 12) = 10.13, *P = 0.0049*), PDGFRβ (F (3, 12) = 4.971, *P = 0.0401*), Claudin-5 (F (3, 12) = 7.257, *P = 0.0107*) and Occludin (F (3, 12) = 15.23, *P = 0.0001*) in the hippocampi. Compared with the MMI group, rats in the MMI + MOX group showed decreased expression of GFAP (F (3, 12) = 9.541, *P = 0.0011*), and increased expression of CX43 (F (3, 12) = 10.13, *P = 0.0020*), PDGFRβ (F (3, 12) = 4.971, *P = 0.0196*), Occludin (F (3, 12) = 15.23, *P = 0.0020*), and Claudin-5 (F (3, 12) = 7.257, *P = 0.0207*). Rats in the MMI + ORC group showed increased expression of CX43 (F (3, 12) = 10.13, *P = 0.0052*) and Occludin (F (3, 12) = 15.23, *P = 0.0144*), and decreased expression of C3 (F (3, 12) = 6.011, *P = 0.0099*). Additionally, there was no significant change in ZO-1 (F (3, 12) = 2.750, *P = 0.0889*) and S100A10 (F (3, 12) = 0.5174, *P = 0.6782*) expression among groups (Figures 3D–L).

### 3.6 Effect of moxibustion on the expression of GFAP, CX43 and colocalization ratio in the region of the vascular structural unit

**FIGURE 4:**
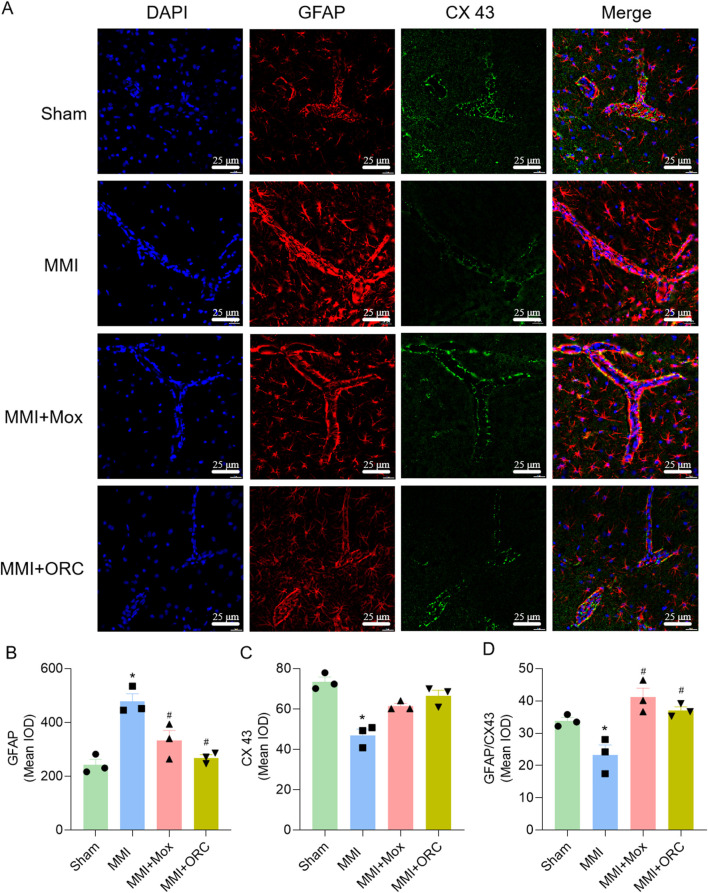
Moxibustion reduced astrocyte activation and increased tight connection between astrocytes. **(A–D)** GFAP and CX43 expression changes in hippocampus at CA1 area by immunofluorescence staining. Scale bar: 50 µm (n = 3) (Tukey’s test or Dunn’s test). Data are presented as means ± SEM. Compared with the Sham group, *P < 0.05. Compared with the MMI group, ^#^ *P < 0.05*.

We also analyzed the colocalization of GFAP and CX43 in CA1 area as an index of astrocytic end-feet function. Compared with the Sham group, GFAP expression in the MMI group was significantly increased (F (3, 8) = 15.88, *P = 0.0011*), CX43 expression was significantly reduced (H = 9.974, *P = 0.0067*), and colocalization of GFAP and CX43 was significantly reduced (F (3, 8) = 11.62, *P = 0.0415*). Compared with the MMI group, GFAP levels in the MMI + MOX (F (3, 8) = 15.88, *P = 0.0196*) and MMI + ORC (F (3, 8) = 15.88, *P = 0.0023*) groups were significant decreased, CX43 levels were significant increased (*P> 0.05*, H = 9.974), and colocalization of GFAP and CX43 was significant increased in MMI + MOX (F (3, 8) = 11.62, *P = 0.0022*) and MMI + ORC groups(F (3, 8) = 11.62, *P = 0.0104*) (Figure 4).

### 3.7 Inhibition of PDGFRβ blocked the effects of moxibustion

**FIGURE 5:**
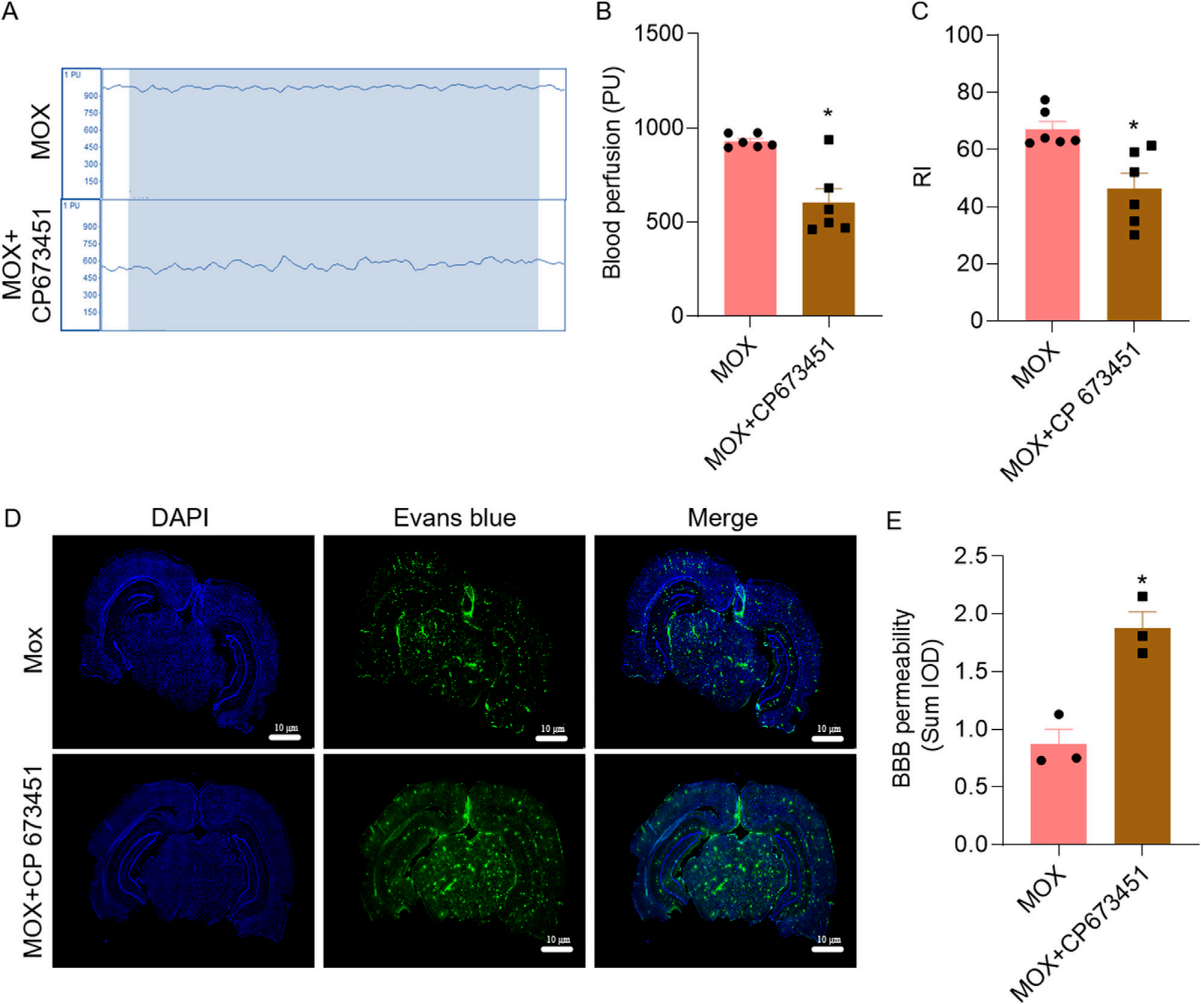
Inhibition of PDGFRβ mitigated the effects of moxibustion on cerebral blood flow, learning and memory, and BBB of VD rats. **(A, B)** Laser Doppler comparison of cerebral blood flow perfusion (n = 5) (*t*-test). **(C)** Recognition index for novel object recognition test (n = 6) (*t*-test). **(D, E)** Evans blue staining for assessing blood-brain barrier permeability (n = 3) (*t*-test). Data are presented as means ± SEM. Compared with the MOX group, *P < 0.05.

Compared with the MOX group, the cognitive index of the rats in the MOX + CP 673451 group significantly decreased (*P = 0.0016*) (Figures 5A–C). Compared with the MOX group, cerebral blood flow in the MOX + CP 673451 group was significantly reduced (*P = 0.0022*), and cerebral blood flow in the MOX + CP 673451 group fluctuated over a wider range (Figures 5A–C)

In order to verify that moxibustion treatment repairs the BBB through GVU, we combined it with the PDGFRβ inhibitor, using the Evans blue permeation rate. Compared with the MOX group, BBB permeability in MOX + CP 673451 group significantly increased, as evidenced by the increase in Evans blue fluorescence (*P = 0.0067*) (Figures 5D, E).

## 4 Discussion

### 4.1 Moxibustion improves cognitive function in VD rats

Moxibustion, as a TCM treatment, has been shown to treat VD with high safety (Liu F. et al., 2023; Kong et al., 2023; Liu et al., 2022), although its mechanism of action is still a current research hotspot (Jiang et al., 2022). In previous reports, moxibustion effectively improved learning and memory of VD rats by inhibiting inflammatory response (Wang et al., 2020; Ruan et al., 2020), reducing neuronal apoptosis (Yang et al., 2021), promoting neuronal vascular regeneration (Zhang et al., 2019), and protecting neuronal mitochondrial damage (Wang et al., 2020). There are many types of animal models to simulate VD, such as the vascular occlusion model (Liu et al., 2019), multiple infarction model (Venkat et al., 2019), thromboembolism model (Ponzer et al., 2023), and the high-fat diet-induced model (Kim and Kim, 2021). However, those models have certain limitations, such as a sharp decrease in cerebral blood flow, increased mortality, and optic nerve damage (Jiwa et al., 2010). In contrast, MMI is more consistent with the clinical symptoms of VD patients (Venkat et al., 2015). Microinfarcts are the most common type of infarct found in VD (Vinters et al., 2000). Cerebral small vessel disease is the most common cerebrovascular lesion in clinically diagnosed VD (Mossanen Parsi et al., 2021). MMI is also causes VD by blocking cerebral small vessel. Based on the above considerations, the MMI model was utilized in this study. To confirm that MMI indeed caused cognitive impairment, we used the positive control drug, ORC in our study as a control (Huang et al., 2017). In fact, ORC did improve the cognitive impairment induced by MMI in rats, indicating the success of the MMI model. In terms of treatment acupoint selection, “Huayu Tongluo” moxibustion at Baihui (GV 20), Dazhui (GV 14), and Shenting (GV 24) can improve learning and memory of VD rats (Gui et al., 2019; Li et al., 2019), and Yang et al. (2021) showed that moxibustion at Guanyuan (RN 4), Mingmen (GV 4) and Dazhui (GV 14) could inhibit neuronal apoptosis and improve cognitive function of VD rats. Guan et al. (2022) demonstrated that moxibustion at Baihui (GV20) and Shenting (GV 24) combined with treadmill running training improved learning and memory of VD rats. Most of those acupoints belong to the Governor Vessel. The Baihui (GV 20), Shenting (GV 24) and Dazhui (GV 14) acupoints adopted in this study also locate on the Governor Vessel, and the three acupoints can be used together to activate blood circulation and remove blood stasis (Wang et al., 2023). In our study, we found that MMI significantly impaired learning and memory, while moxibustion at the Governor Vessel acupoints improved learning and memory in VD rats.

### 4.2 GVU may be an important mechanism for moxibustion to regulate cerebral blood flow and BBB permeability in VD rats

With the in-depth study of VD, more and more evidence shows that vascular lesions and vaso-related risk factors are the main pathogenesis of VD (Li et al., 2021; Agrawal and Schneider, 2022). The regulation of GVU is considered to be an essential factor in maintaining normal cerebral vascular function (He and Duan, 2023). GVU is a structure composed of neurovascular units, which emphasizes the central role of glial cells, such as the interaction between astrocytes, microglia, and perivascular cells involved in the regulation of cerebral blood flow, BBB formation, and removal of toxic substances (Wu et al., 2023). Moxibustion can protect the structural and functional integrity of BBB (Zhou et al., 2019). Liu S. et al. (2023) showed that moxibustion regulated the complex aquaporin-4 polarization of perivascular astrocytes and BBB, affected the structure and function of BBB, and thus improved learning and memory of APP/PS1 mice. Our present study further supported the effect of moxibustion on learning and memory, cerebral blood flow and BBB in VD rats. ORC is a newly emerging nootropic drug, and it can improve learning and memory and repair damaged neurons through repairing BBB (Jiang et al., 2023). Moreover, study has shown that ORC can regulate GFAP expression and inhibit neuroinflammation (Youn et al., 2023). Therefore, we selected ORC as a positive control in our study to verify the efficacy of moxibustion in improving learning and memory in VD rats. Interestingly, the effects of moxibustion and ORC were significantly different in terms of ameliorating neuronal degeneration in different regions of the hippocampus, which may be related to the different mechanisms by which they produce their effects.

### 4.3 Moxibustion affects GVU to improve neurological function in VD rats by modulating communication between pericytes and astrocytes

As the largest type of glial cells in GVU, astrocytes participate in almost all CNS functions (Sofroniew, 2020). They play a very important role in neuronal support, immune regulation and BBB regulation, and synaptic plasticity (Almad et al., 2022). GFAP is a structural marker of astrocytes and a common marker of astrocyte activation (Jurga et al., 2021). Astrocytes have two main cell morphology and phenotypes after being activated, which are neurotoxic or proinflammatory phenotype (A1) and neuroprotective against neurotoxic or anti-inflammatory phenotype (A2) (Liddelow et al., 2017). C3, a marker of the A1 phenotype, and S100A10, a marker of the A2 phenotype, are often used to test the expression of astrocytes after polarization (Hwang et al., 2022). Astrocytes form highly coupled intercellular networks in the CNS through gap junctions and semi-channels, and CX43 is the main connexin in astrocytes. As an important part of GVU, PDGFRβ is a specific marker of pericyte. It is also crucial for promoting angiogenesis and maintaining the integrity of the BBB structure (Cicognola et al., 2023). As tight junction proteins between brain endothelial cells, Occludin, Claudin-5 and ZO-1 are essential for maintaining the structural and functional integrity of GVU and BBB (Wu et al., 2023). Inhibition of CX43 expression leads to serious learning and memory impairment (Tao et al., 2021). CX43 is an essential messenger in the information network between astrocytes, and the stability of CX43 also determines the distribution and expression of other membrane proteins, lipids and cytoplasmic connectivity components, and CX43 provides a stable framework for the activity and function of astrocytes (Cibelli et al., 2021). The results of our experiment showed that moxibustion treatment reduced GFAP and increased CX43 expression. They also showed that moxibustion treatment on acupoints in the Governor Vessel reduced the activation of astrocytes and increased tight junctions among glia, pericyte and endothelial cells. Interestingly, in regulating GVU, OCR exhibited effects similar to moxibustion. Although the mechanism by which OCR improves cognition is thought to be related to the regulation of neurotransmitters, particularly acetylcholine, there is also report indicating that OCR can ameliorate BBB damage in ischemic stroke model (Huang et al., 2017).

To further validate our results that moxibustion acts through the GVU to reduce BBB destruction and thereby improve neurological function in VD rats, we used CP 63451 (Lane et al., 2022), a PDGFRβ inhibitor. We found learning and memory impairment and BBB disruption as well as reduced cerebral blood flow in the MOX + CP 673451 group, suggesting that amelioration of neurological dysfunction in the VD rats by moxibustion could act by affecting the GVU, and influencing the expression of pericyte PDGFRβ, which in turn regulates the expression of astrocytes and related gap junction proteins. Astrocyte-pericyte crosstalk, as well as other components of the GVU is important to maintain normal BBB function (Girolamo et al., 2021). Therefore, the results of the present study suggest that moxibustion may be able to repair BBB function by promoting the astrocyte-pericyte crosstalk and thus improving neurological function of VD rats.

### 4.4 Shortcomings and prospects

This study only investigated the mechanism of moxibustion for improving learning and memory of VD rats by regulating CX43, but did not verify whether moxibustion affected GVU and the whole pathological process of VD through CX43. Whether moxibustion can exert its effect by regulating other molecular mechanisms related to GVU is also a question worth exploring in the future.

## 5 Conclusion

In conclusion, moxibustion at GV acupoints promotes cerebral blood flow and repairs BBB function in rats with MMI, likely through repairing GVU. Our study could provide a possible experimental basis for the treatment of VD with moxibustion in clinical practice.

## Data Availability

The raw data supporting the conclusions of this article will be made available by the authors, without undue reservation.

## Abbreviations

VD, vascular dementia; GVU, glial vascular unit; BBB, blood-brain barrier; MMI, multiple microinfarct; GFAP, glial fibrillary acidic protein; CX43, Connexin 43; PDGFRβ, platelet derived growth factor receptor β; ZO-1, Zonula occludens-1.
