# Supplementary material for: Exploring the effects of moxibustion on cognitive function in rats with multiple cerebral infarctions from the perspective of glial vascular unit repairing
Source: Front Pharmacol. 2024 Oct 22;15:1428907. doi: 10.3389/fphar.2024.1428907 (PMC11539022; doi:10.3389/fphar.2024.1428907)

Supplemental data

Original blots for the Figure.3D

GFAP (GFAP-2 was the representative blot in figures)


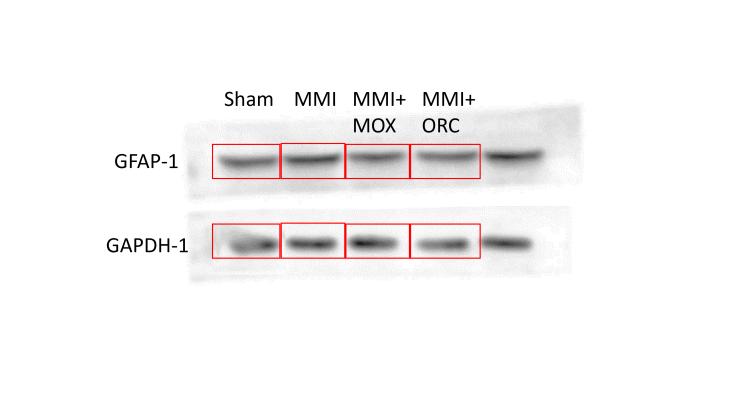

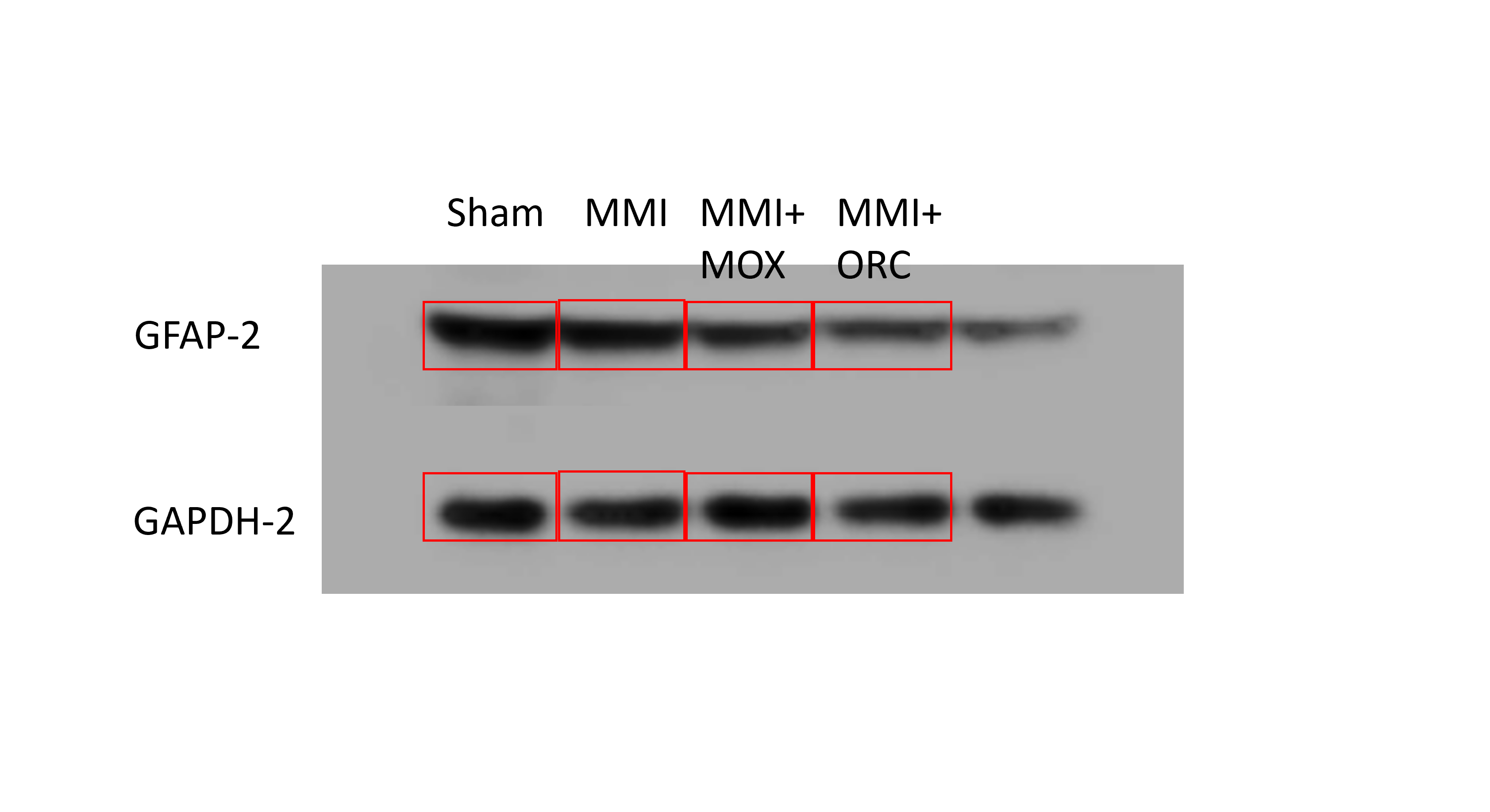


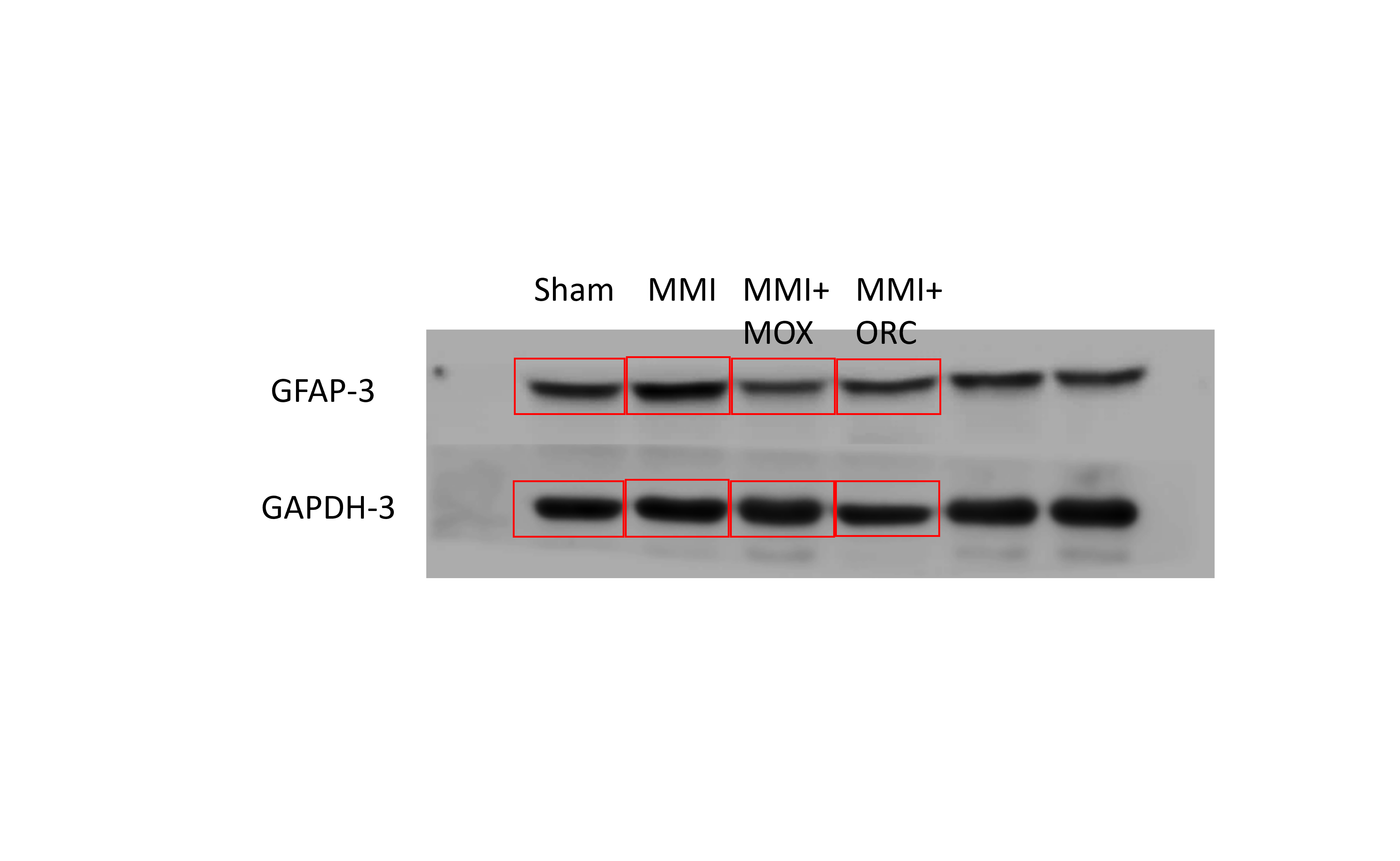

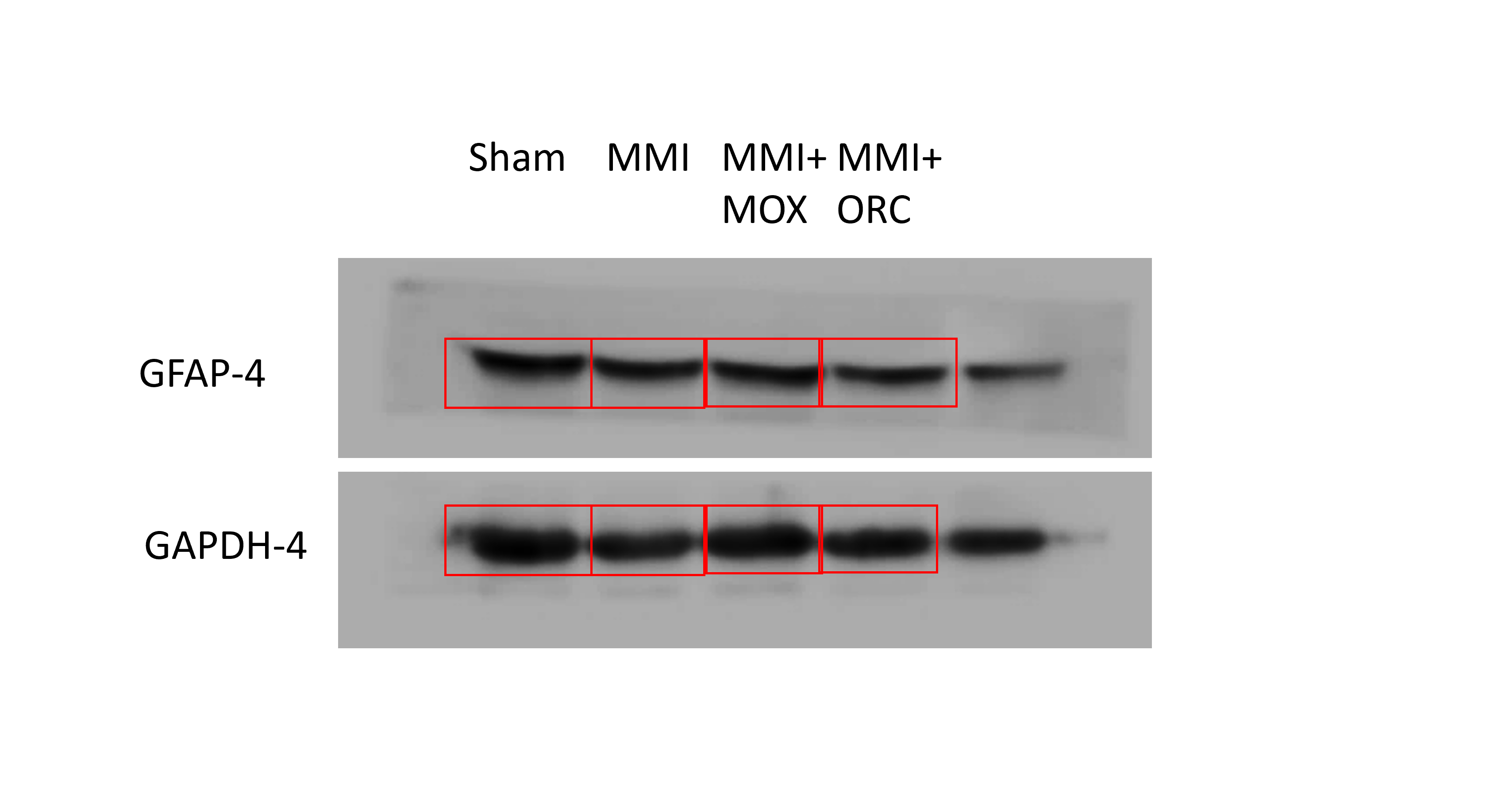


CX 43 (CX 43-1 was the representative blot in figures)


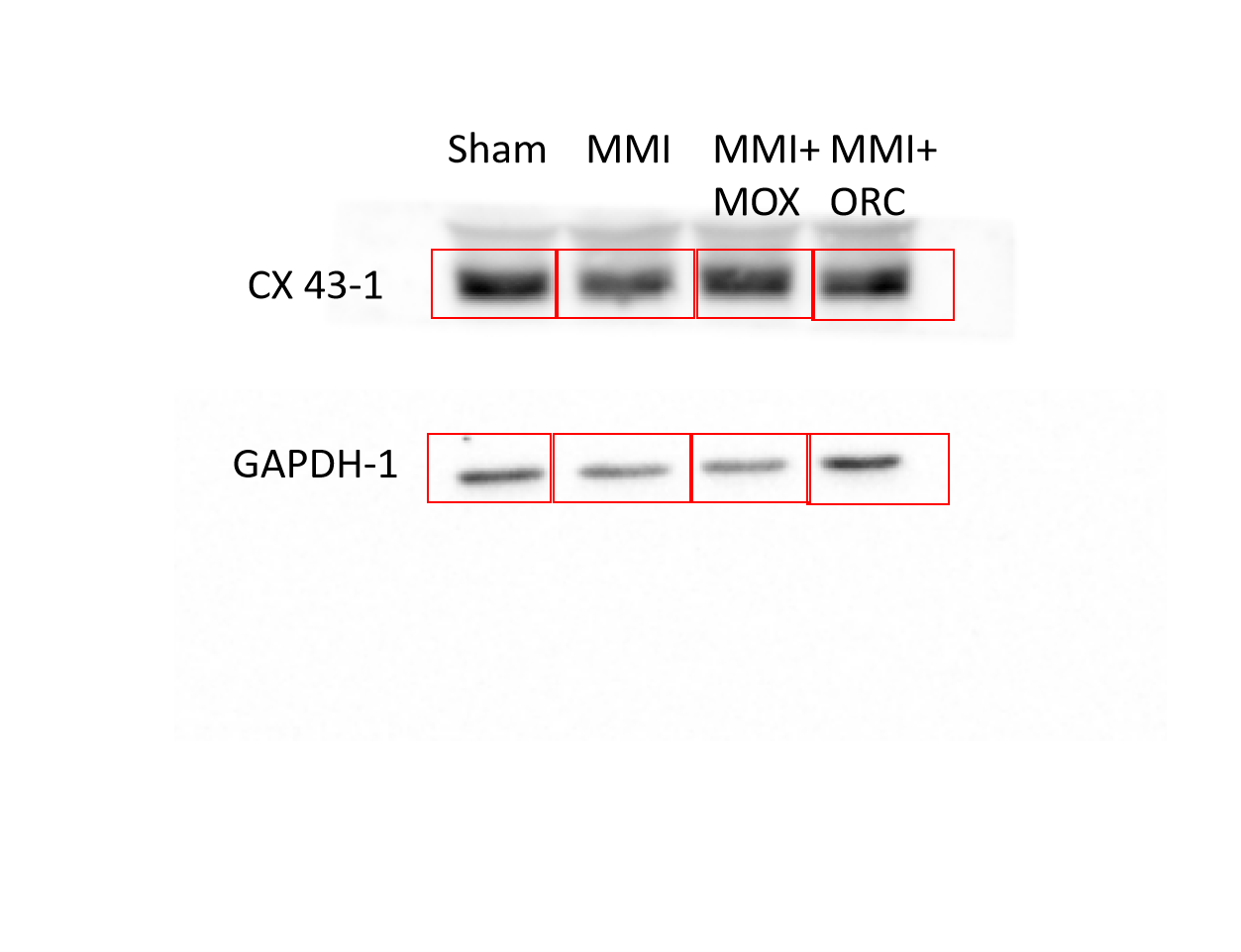

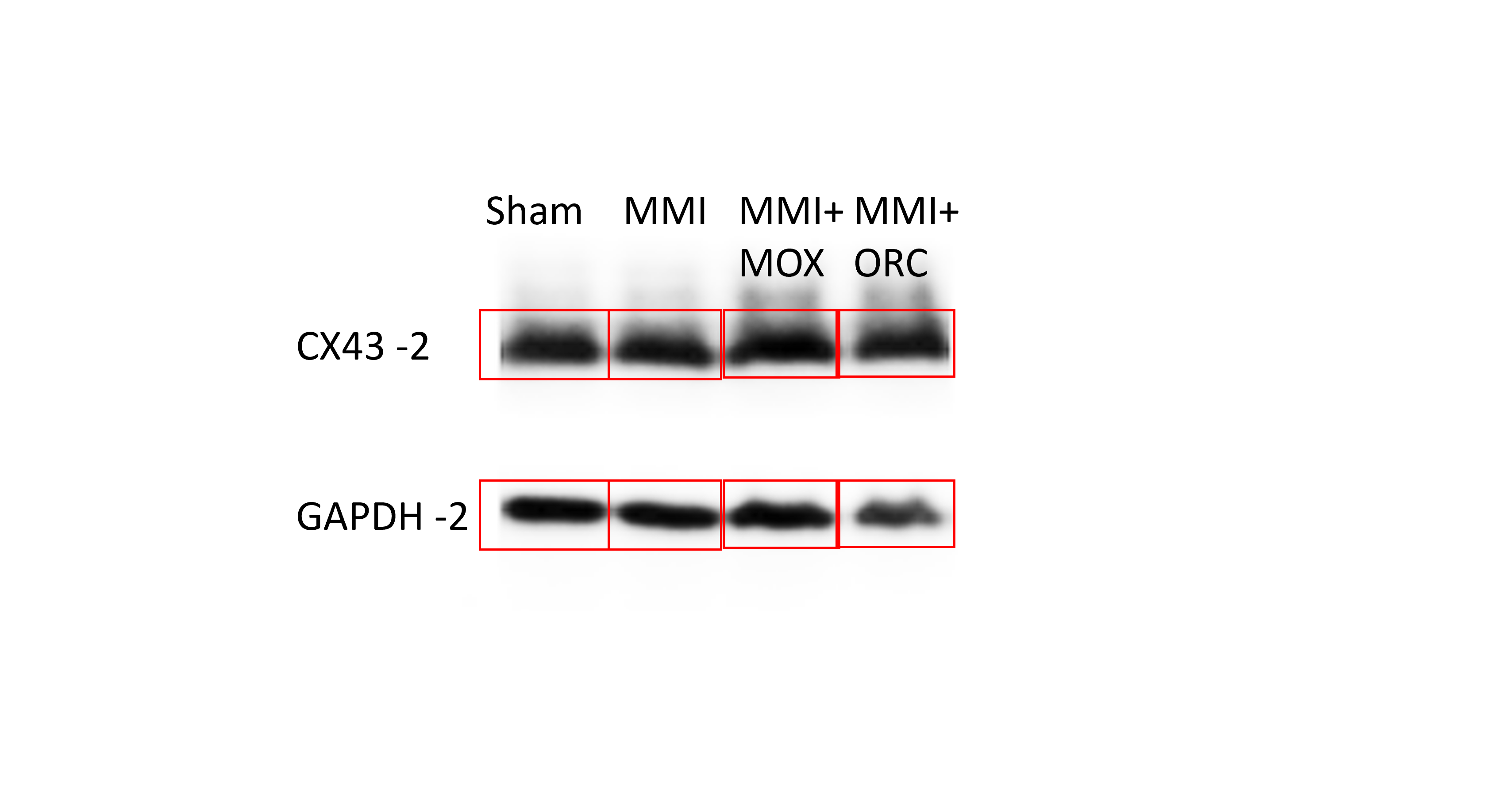


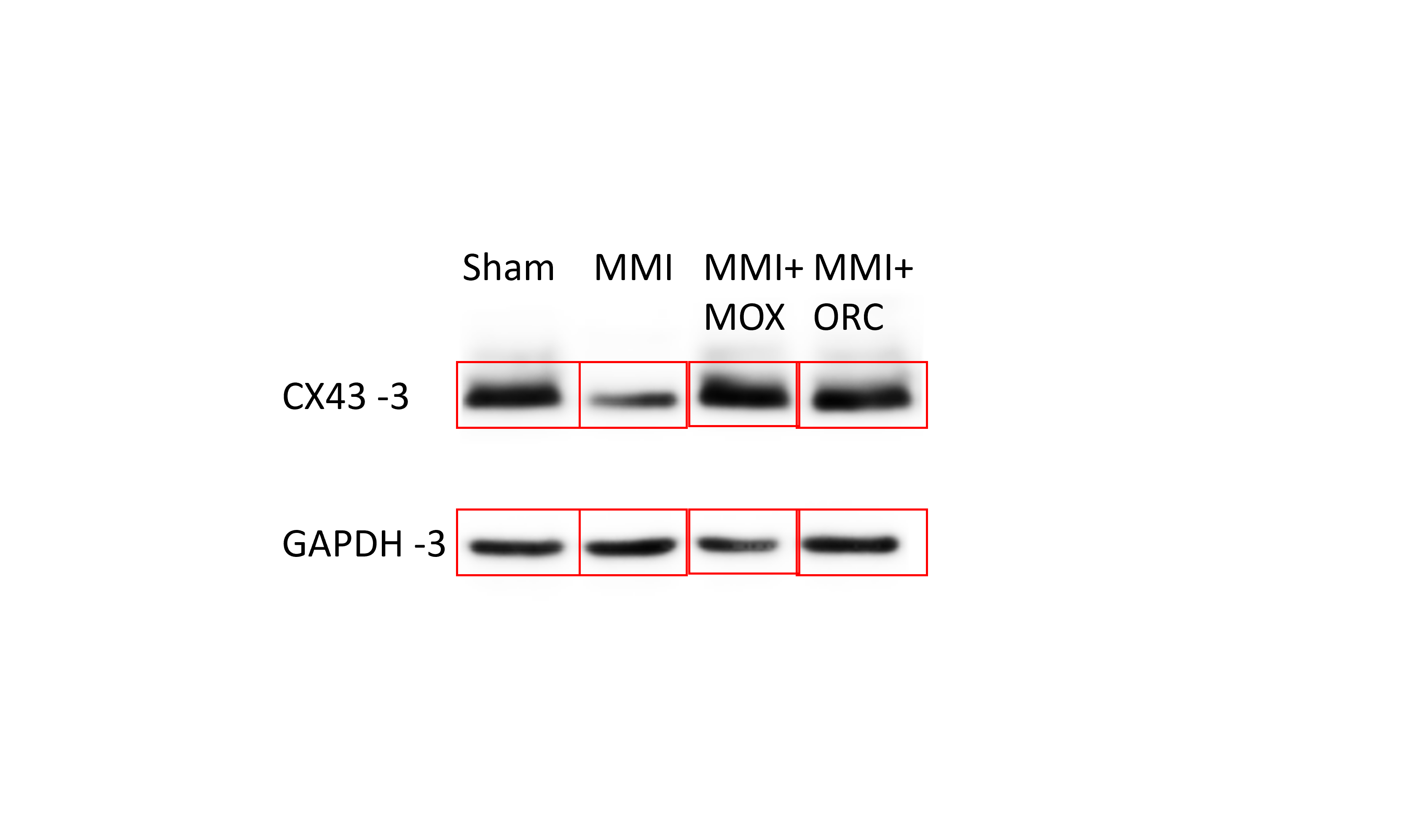

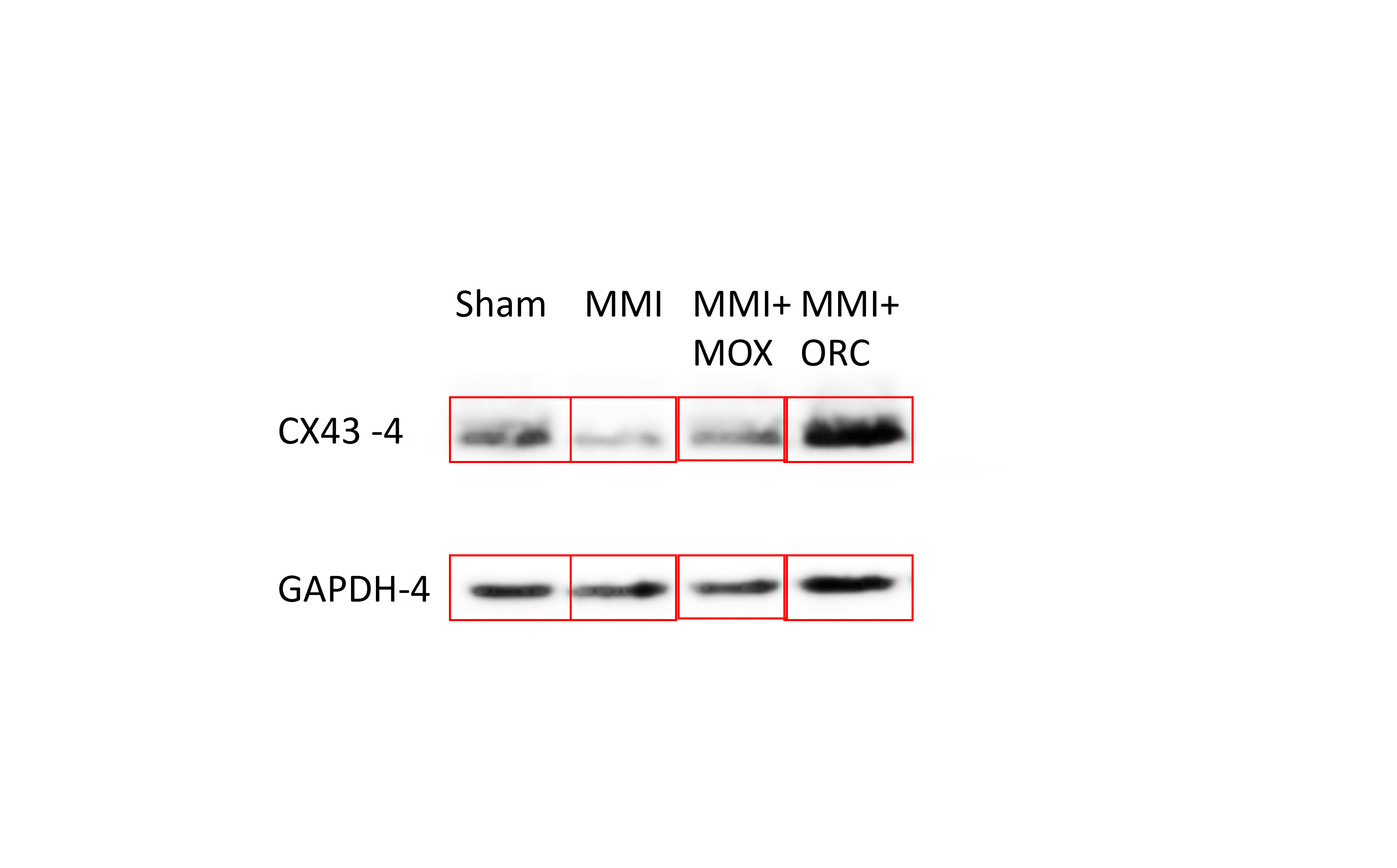


PDGFR β (PDGFR β-2 was the representative blot in figures)


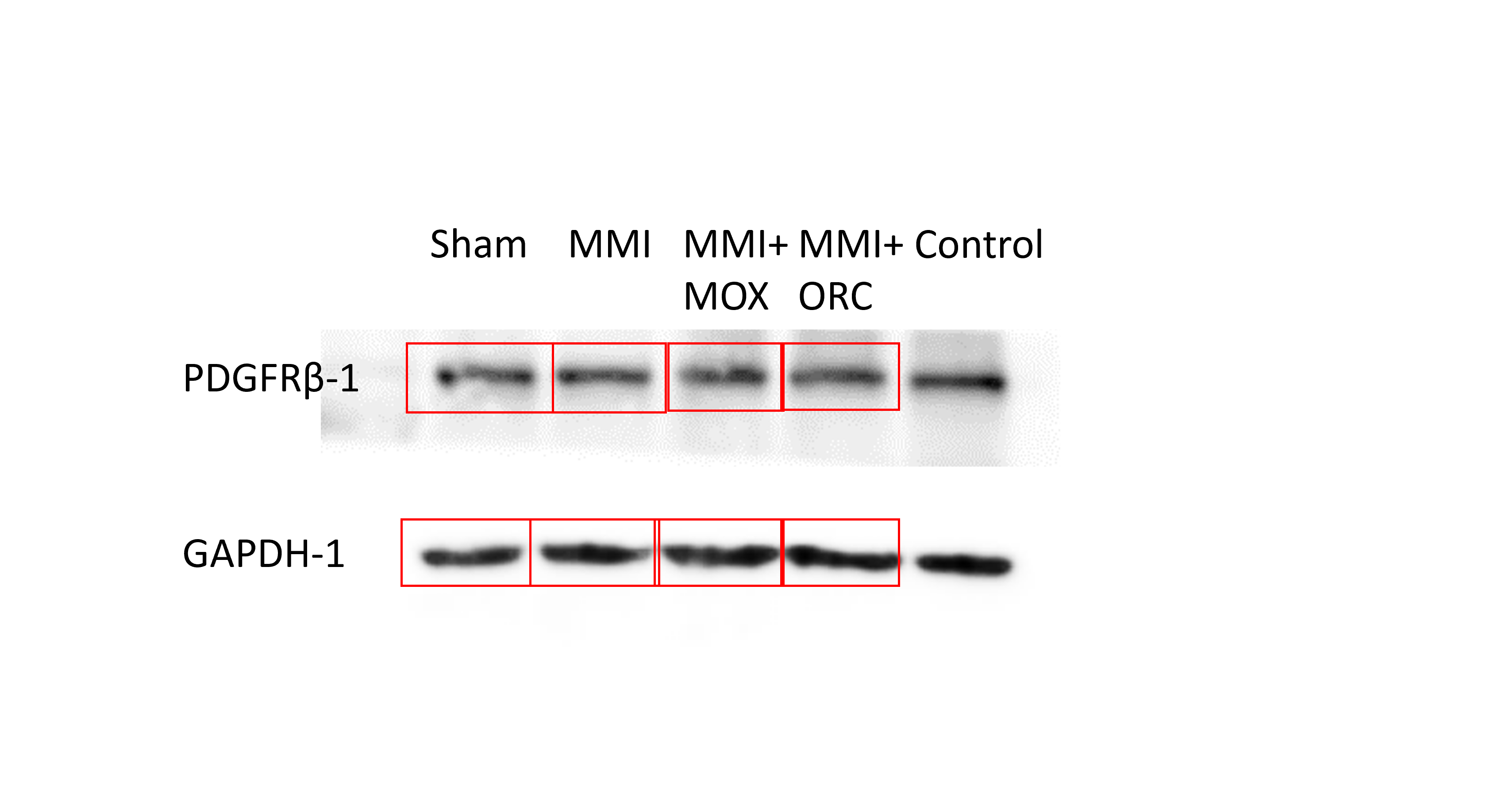

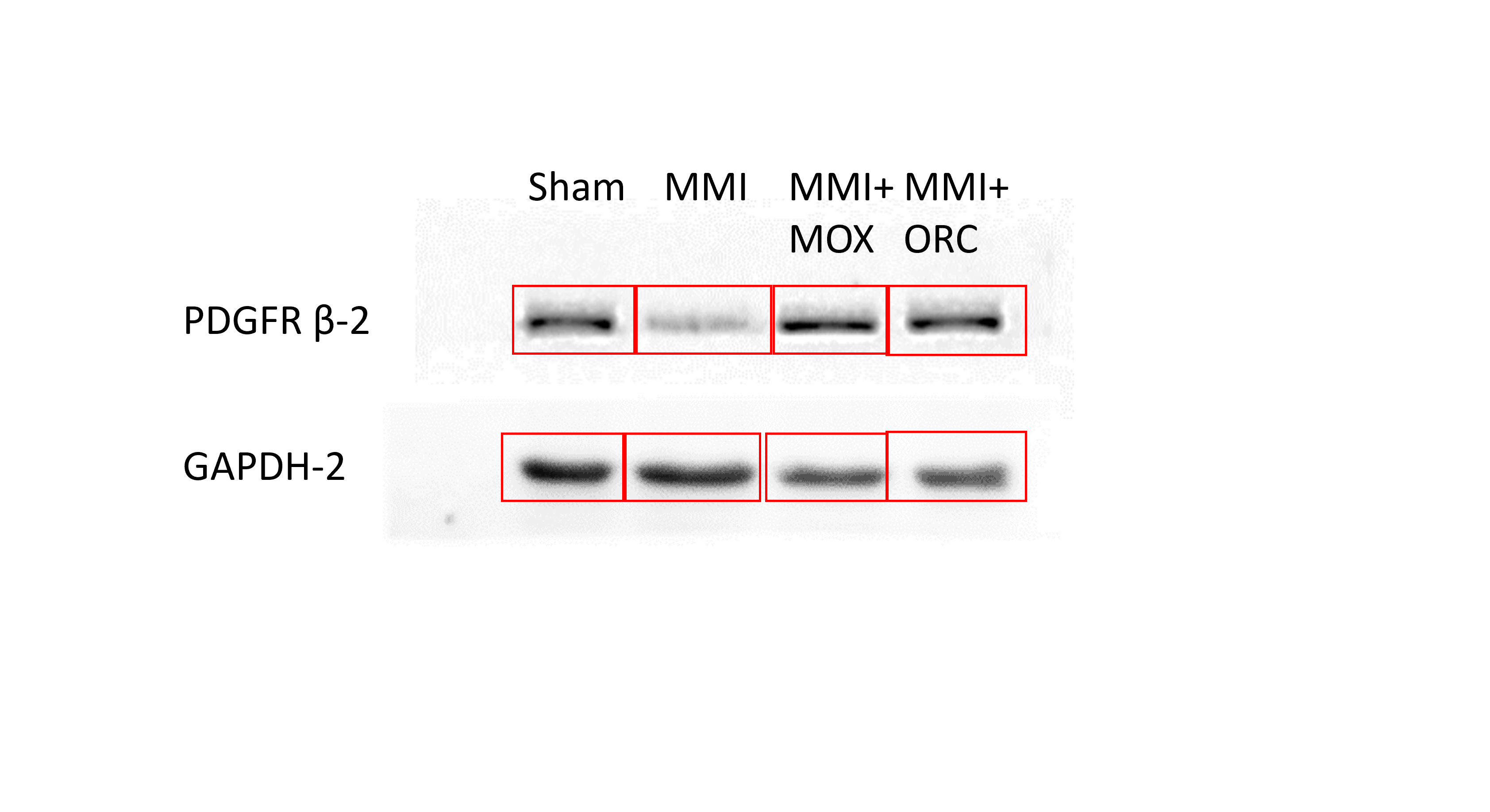


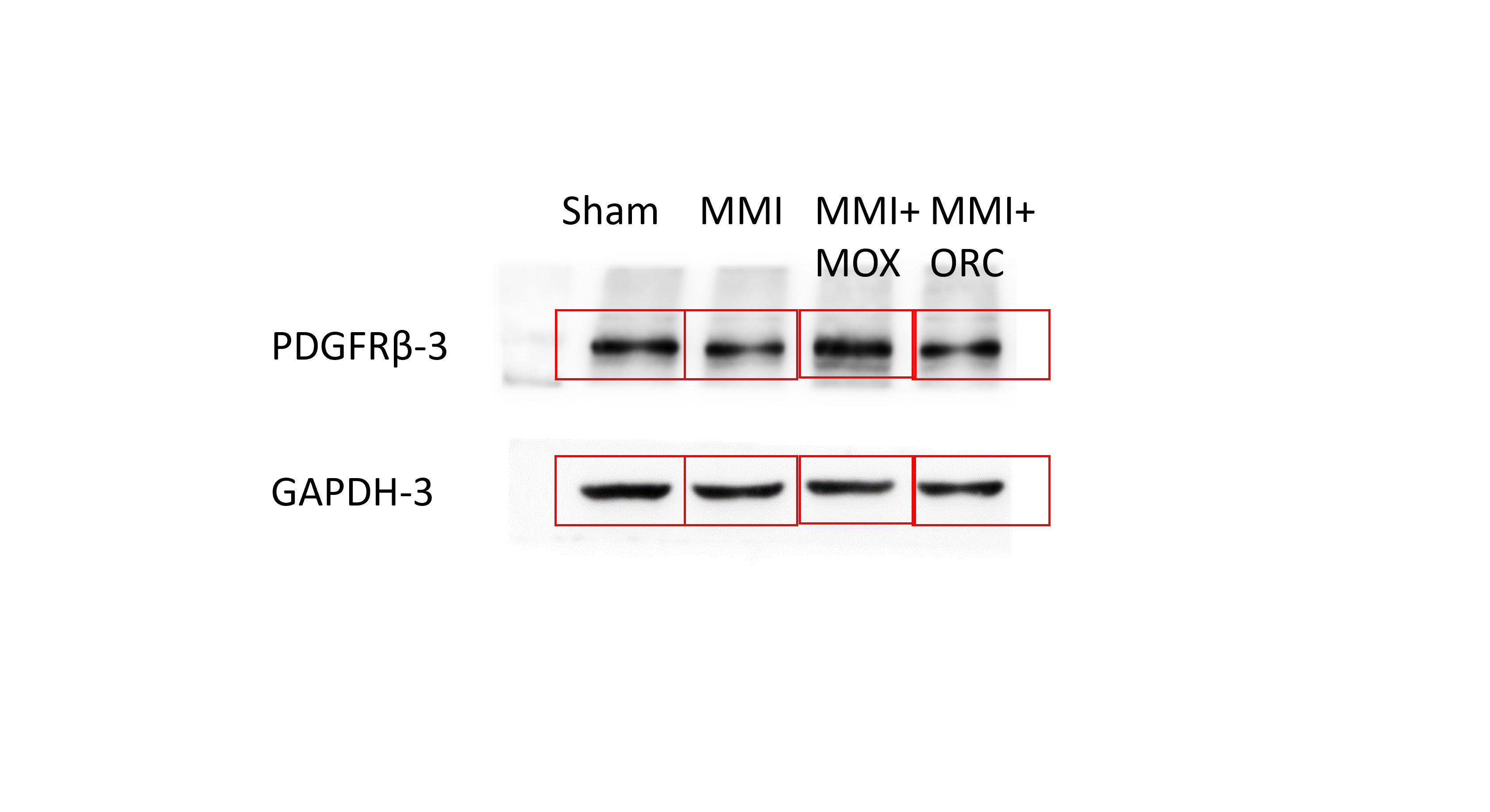

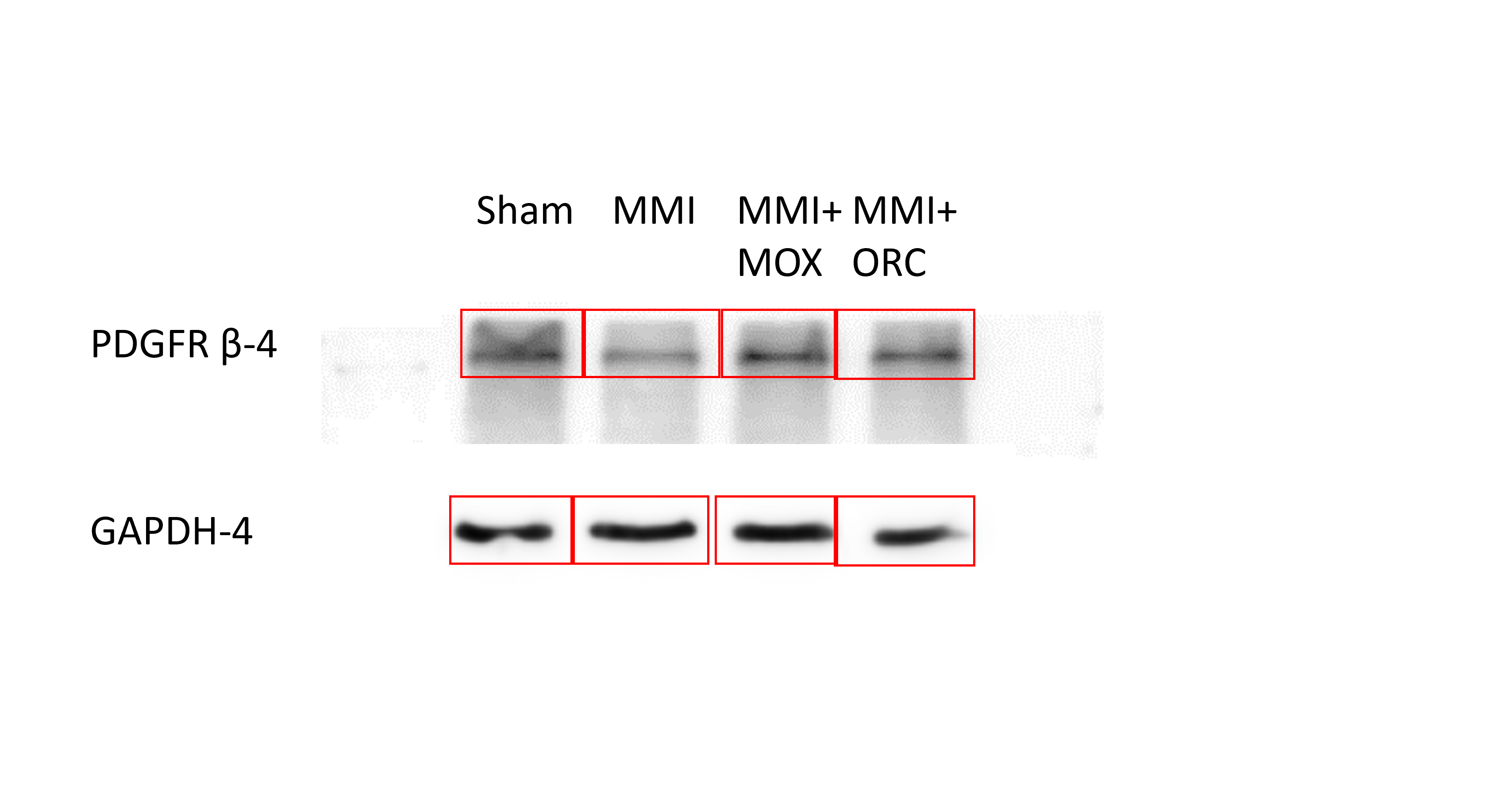


ZO-1 (ZO-1-3 was the representative blot in figures)


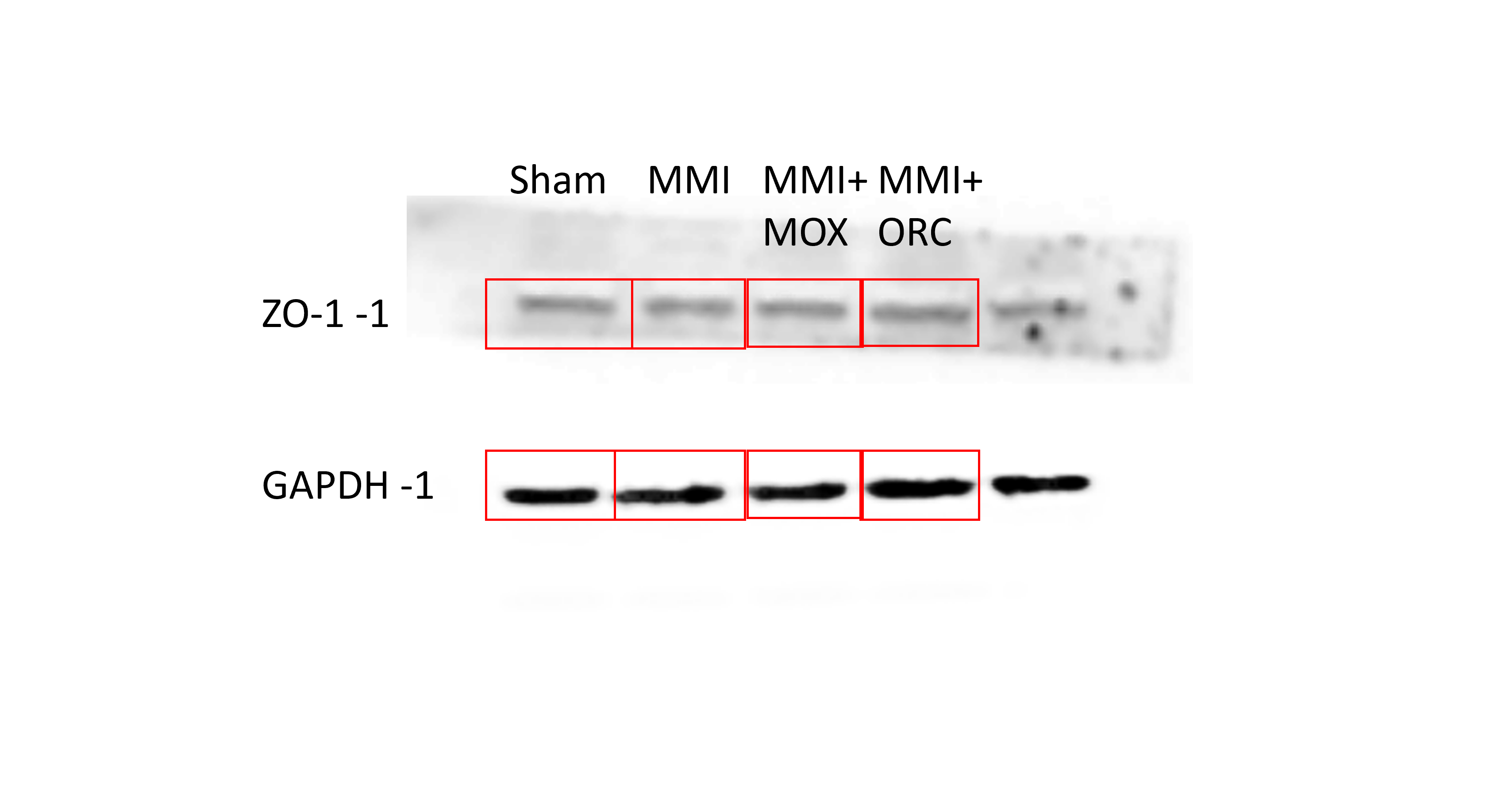

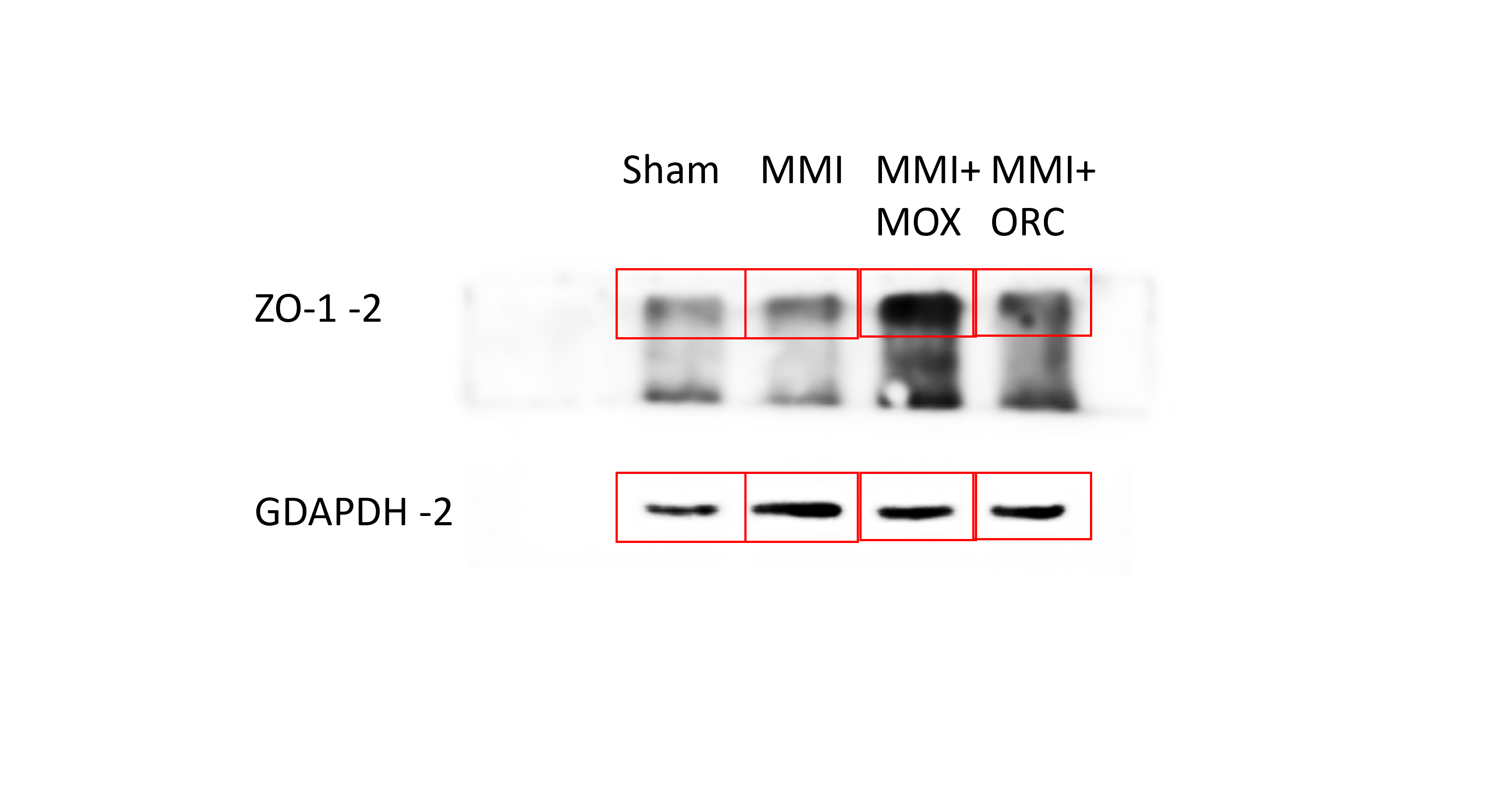


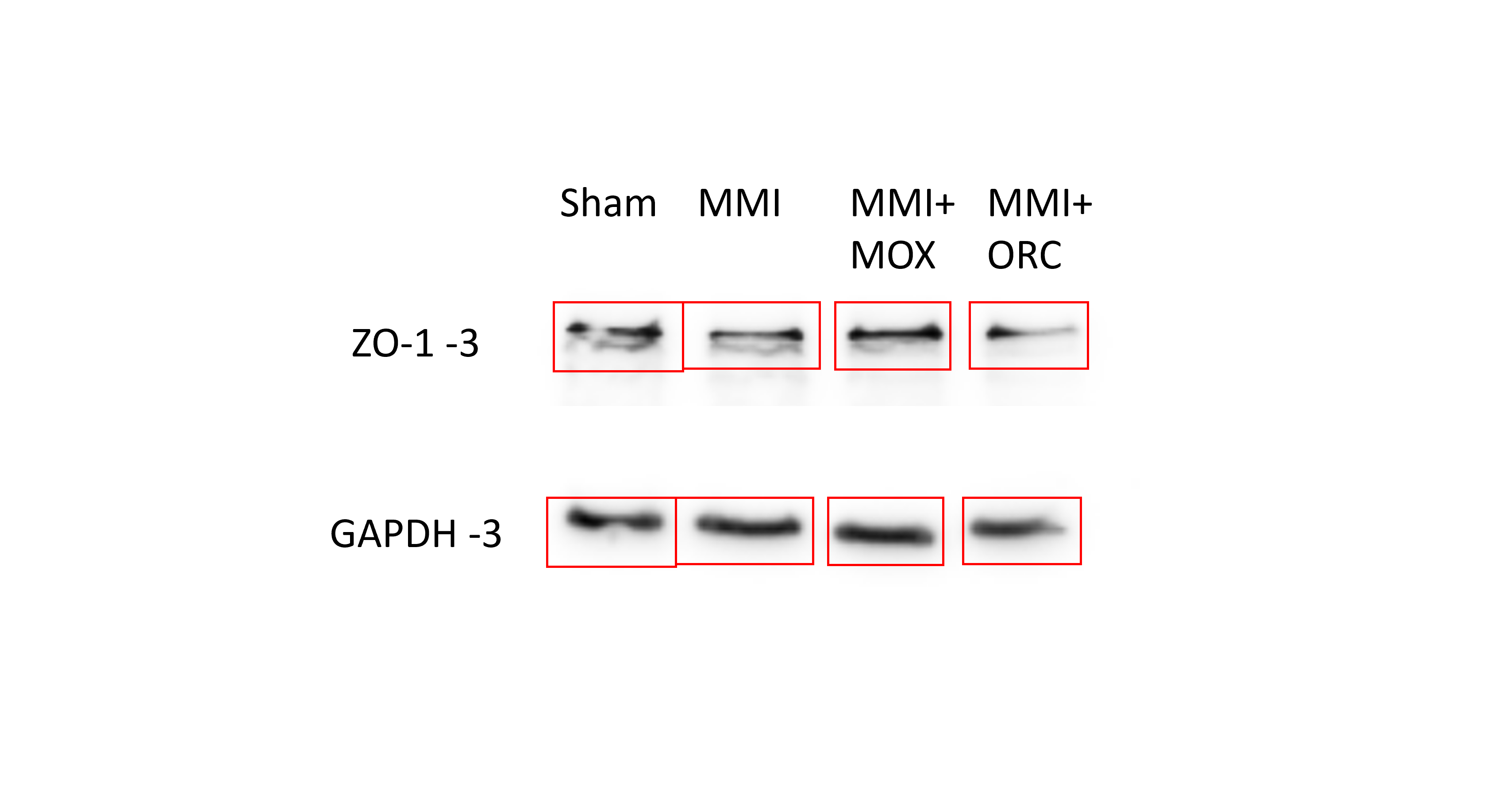

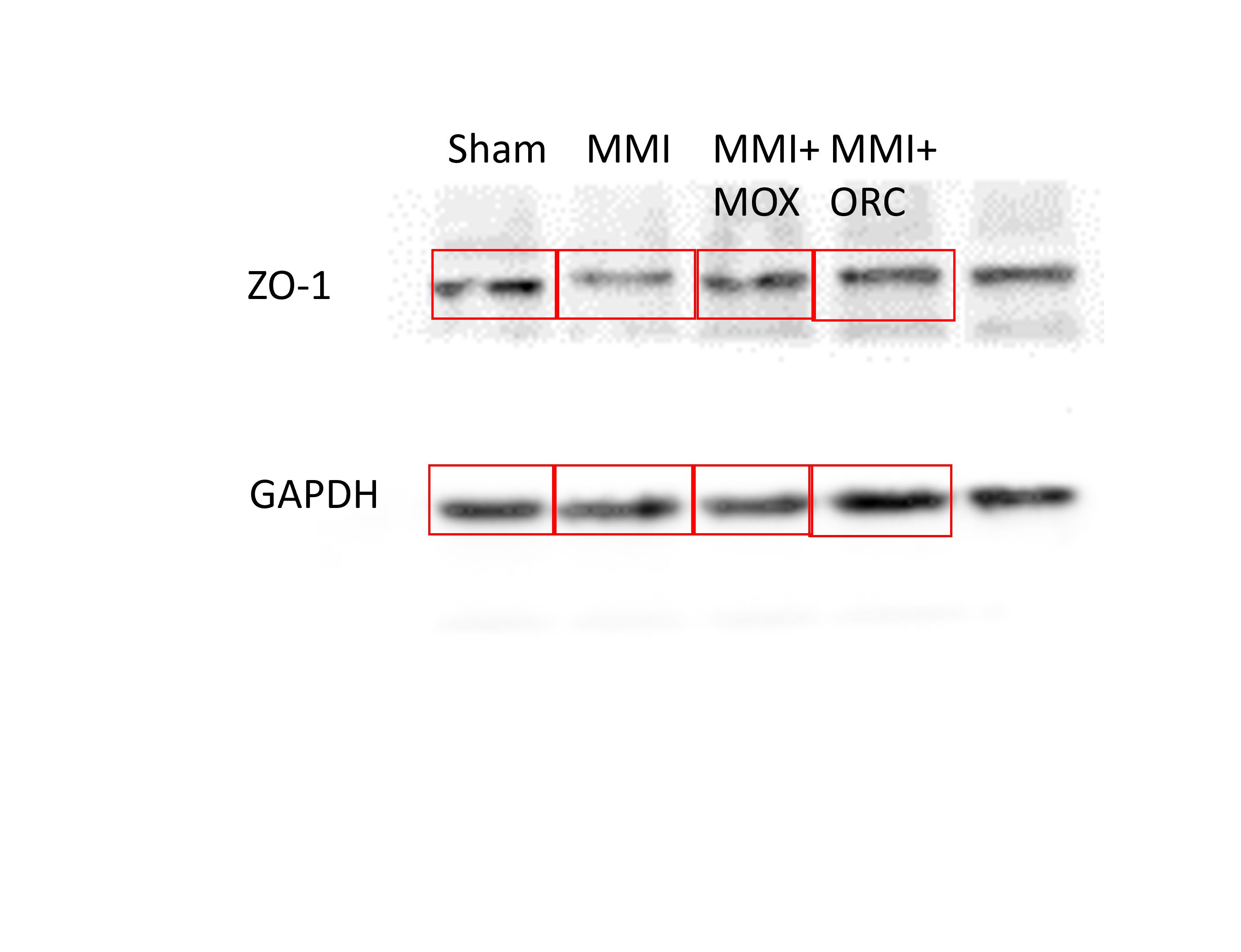


C3 (C3-4 was the representative blot in figures)


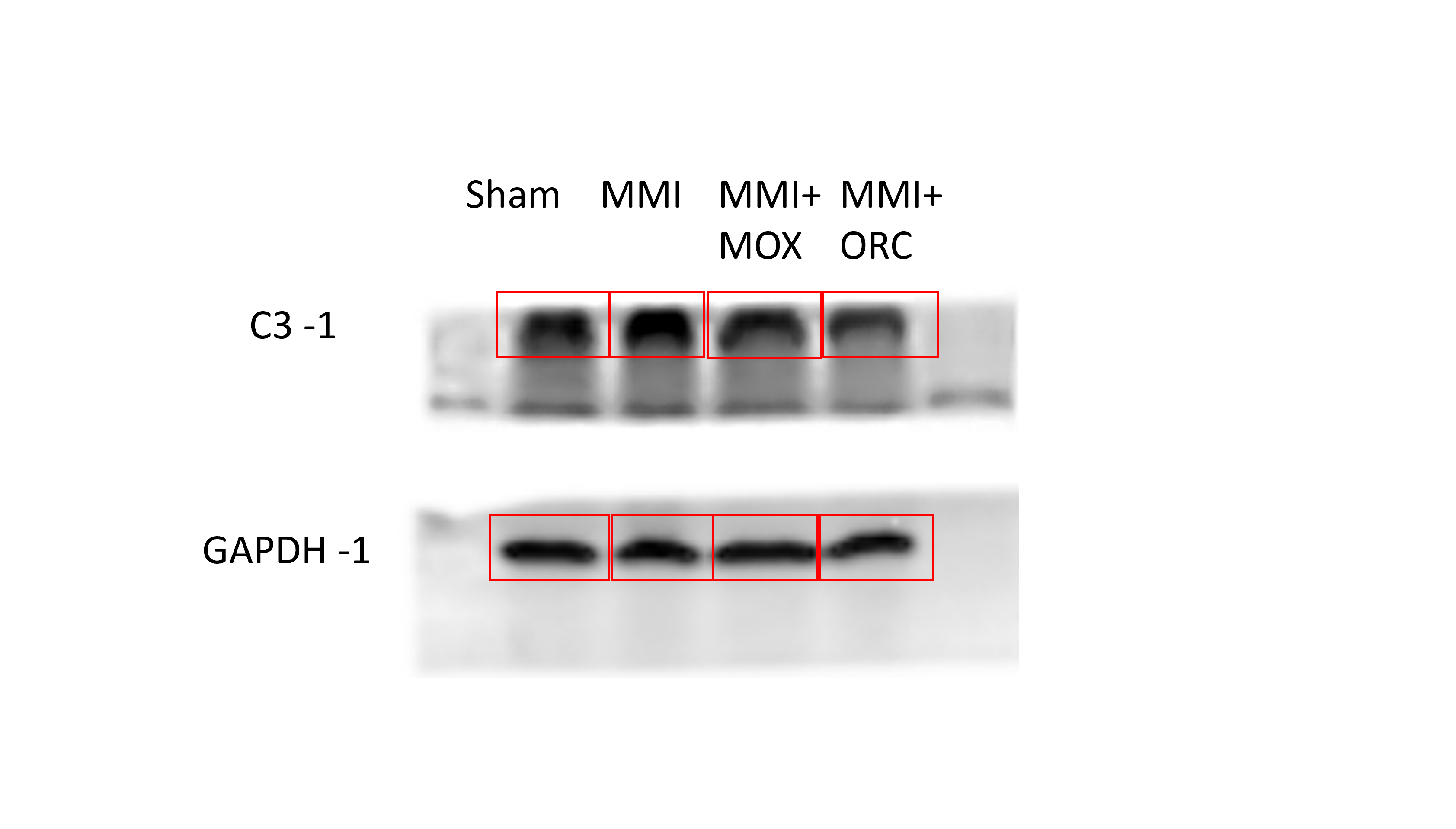

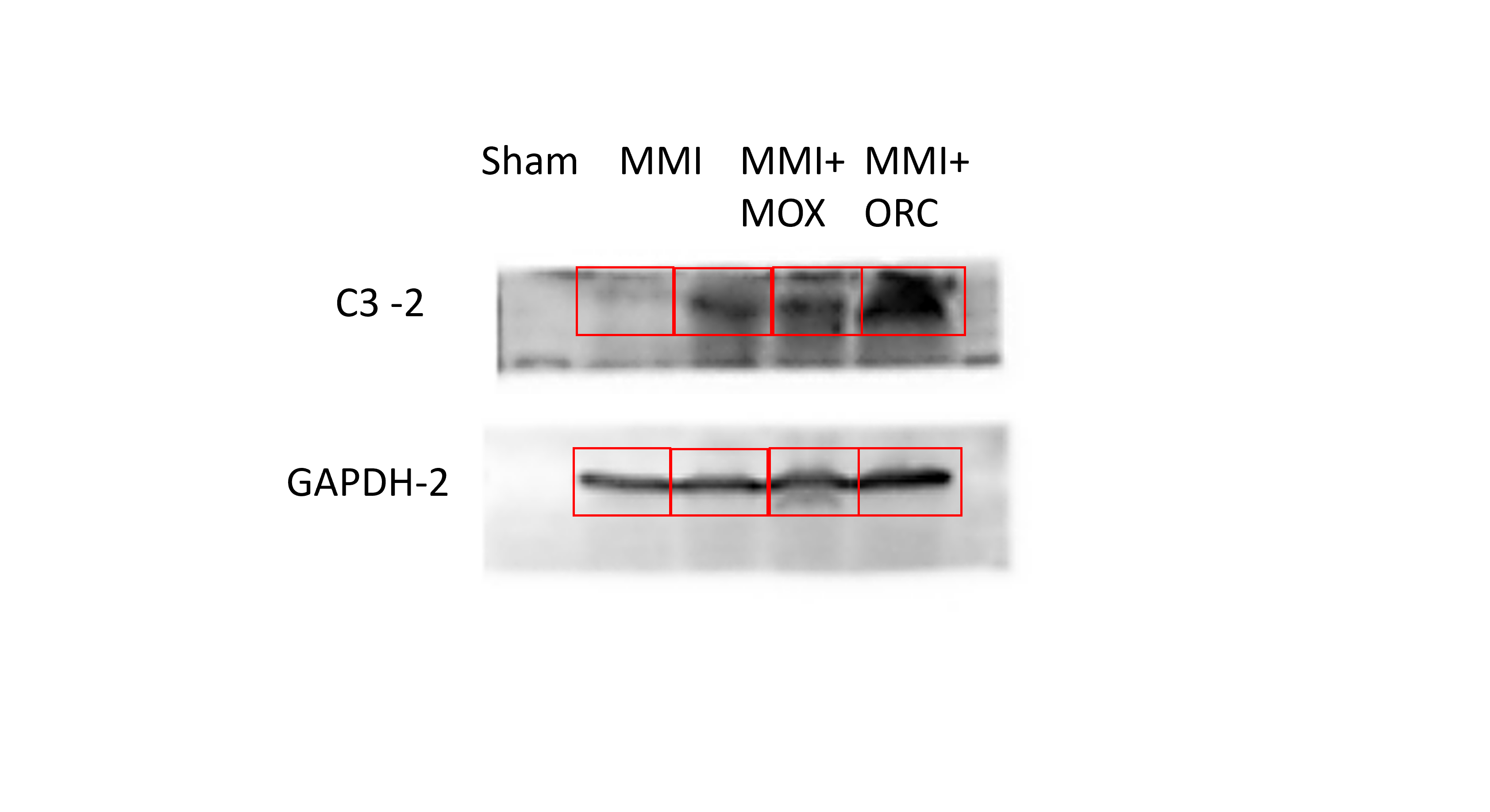


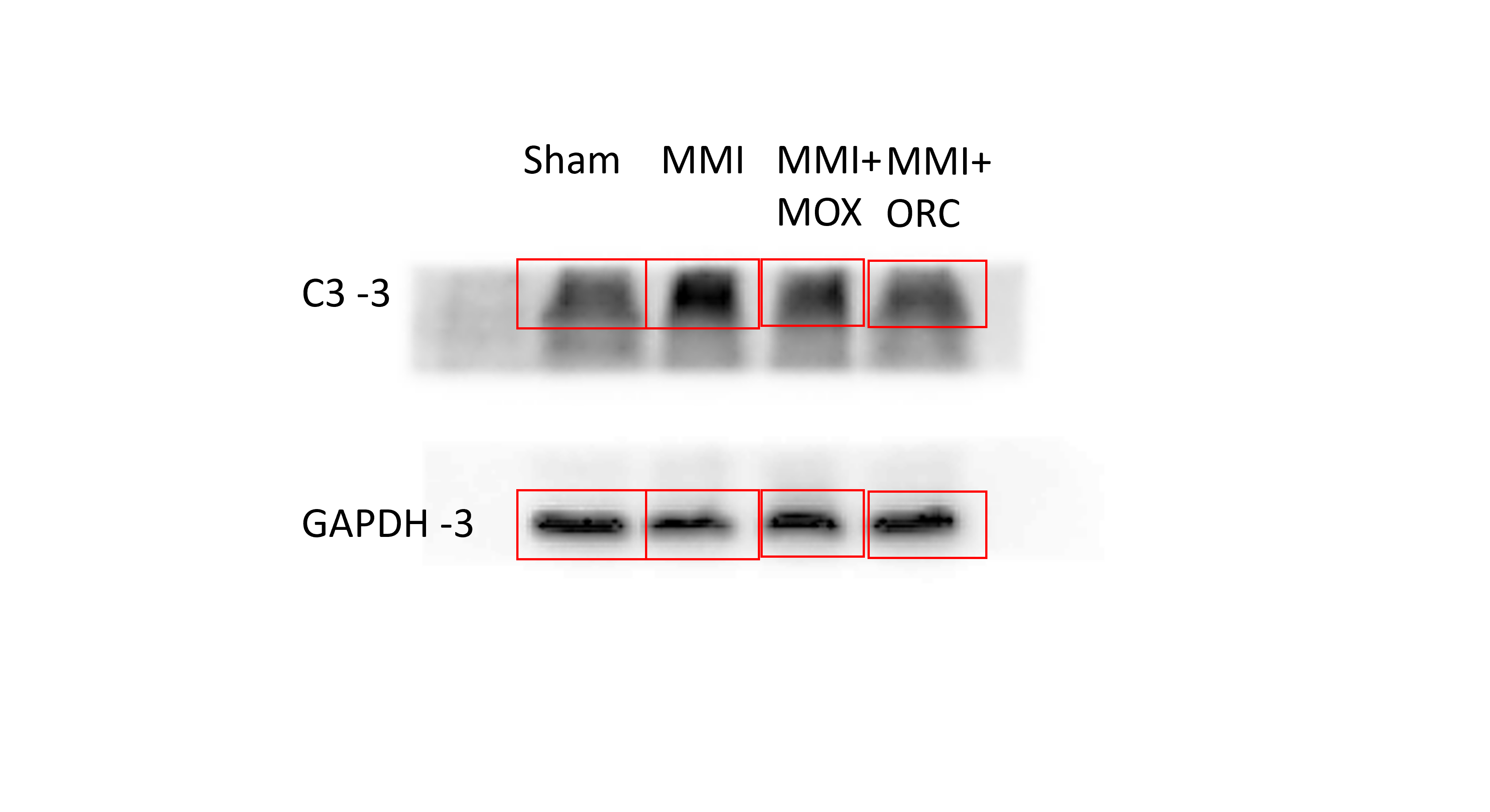

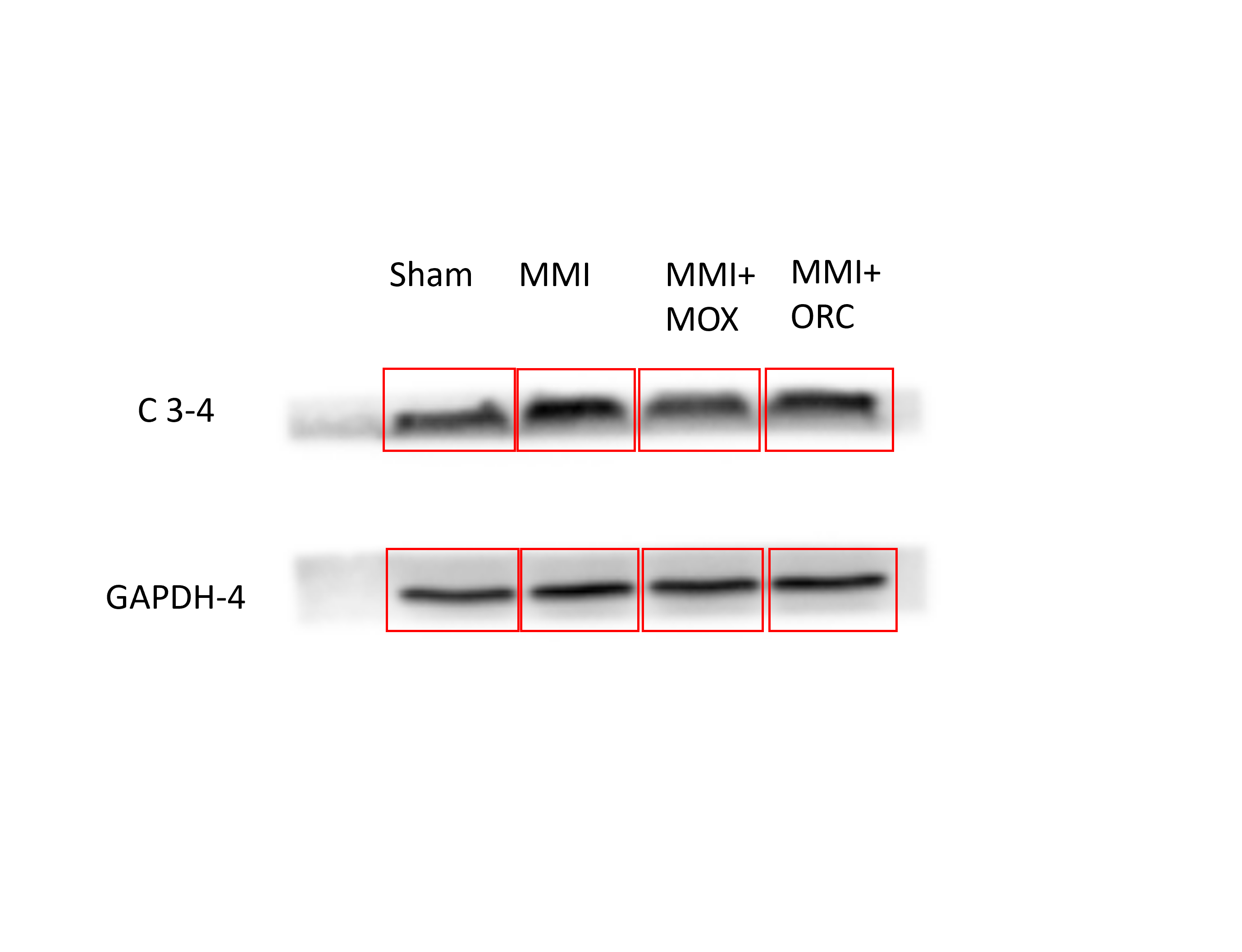


S100A10 (S100A10-4 was the representative blot in figures)


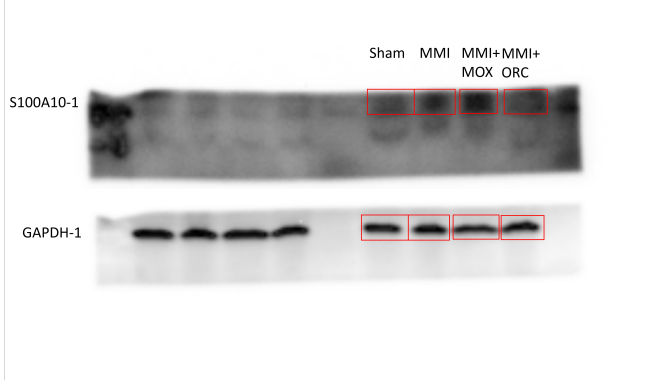

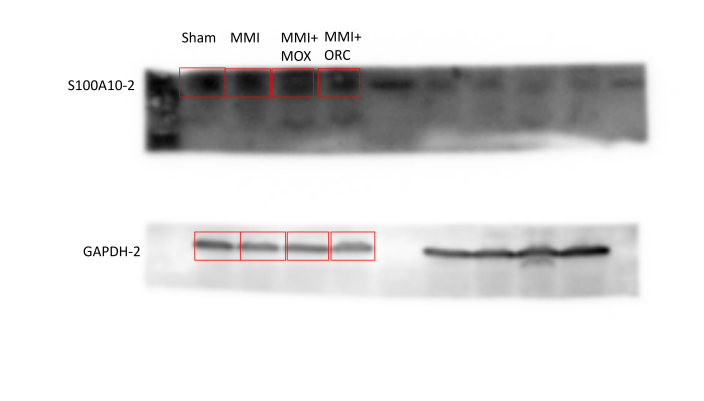


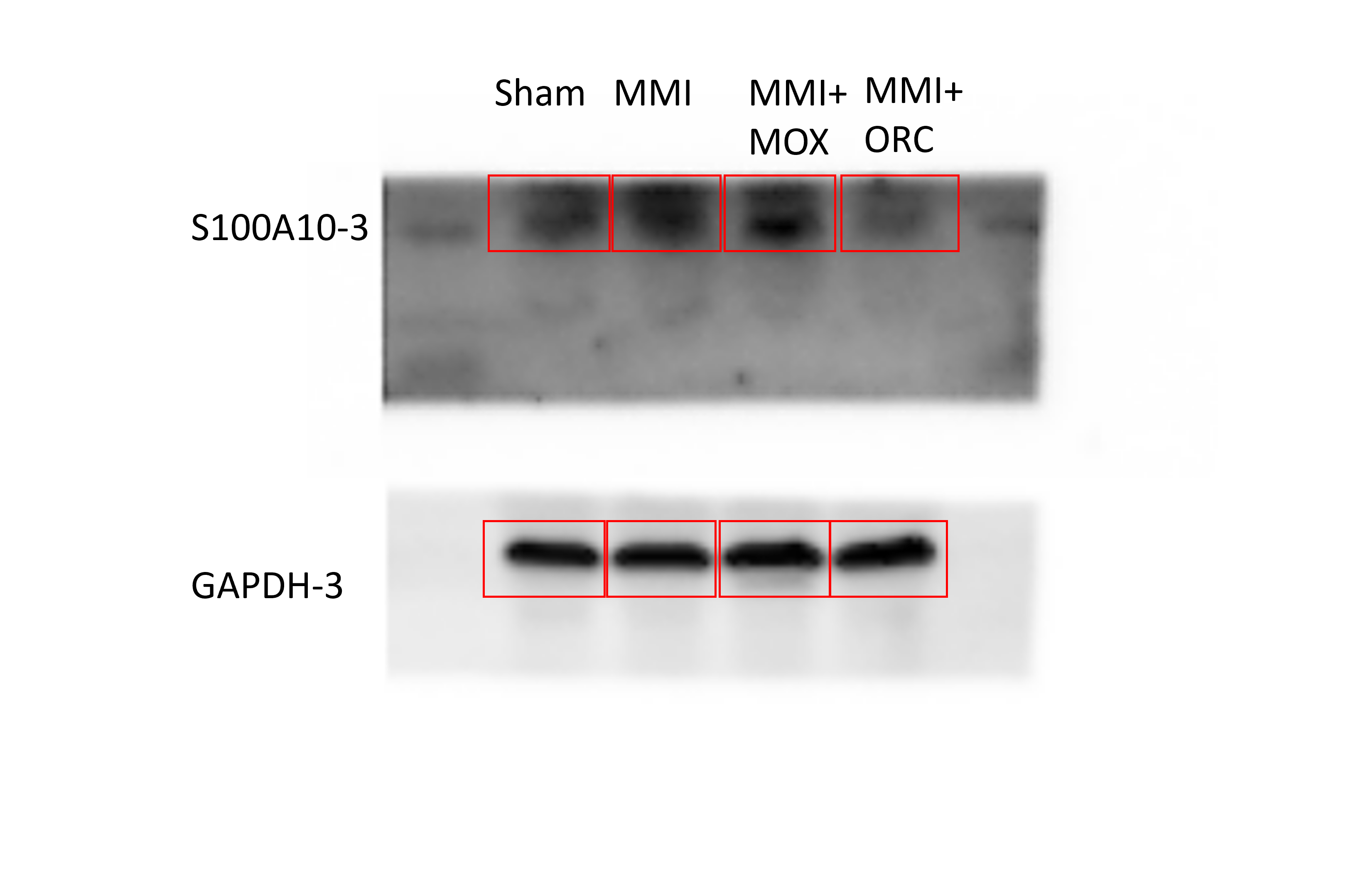

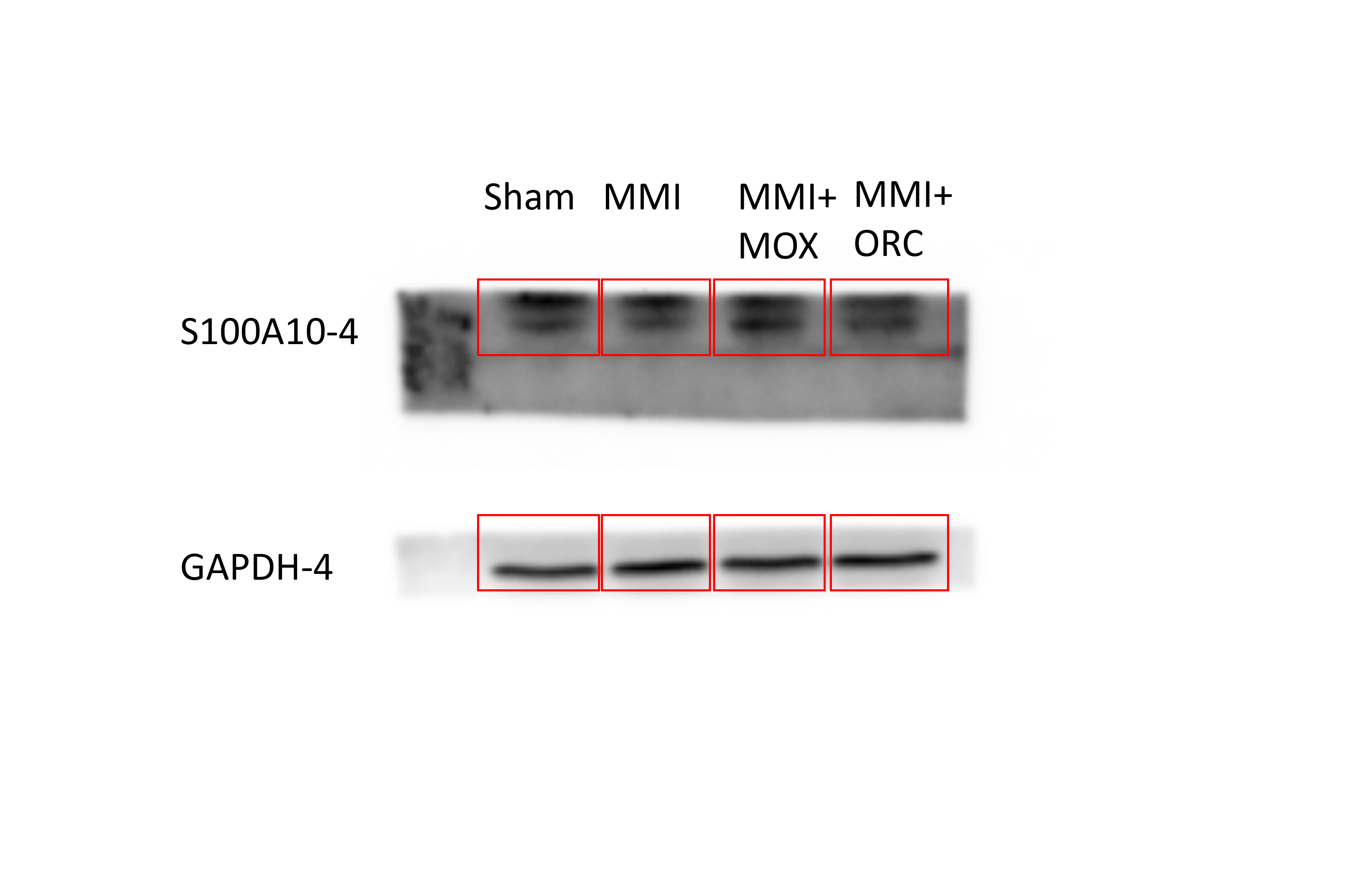


Claudin-5 (Claudin-5-1 was the representative blot in figures)


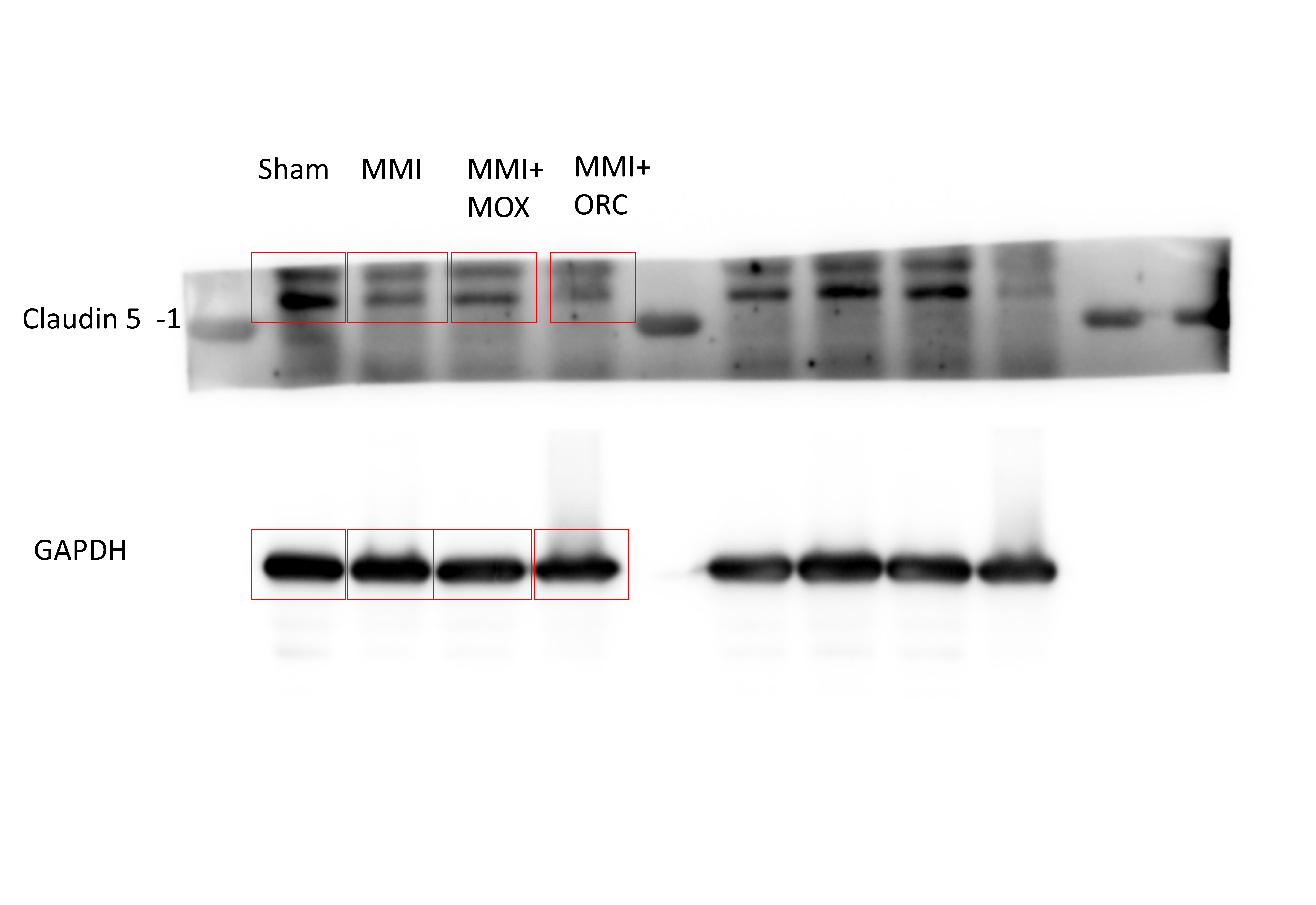

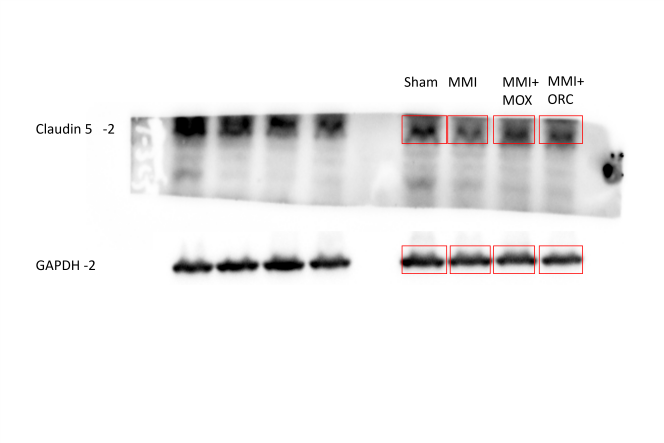


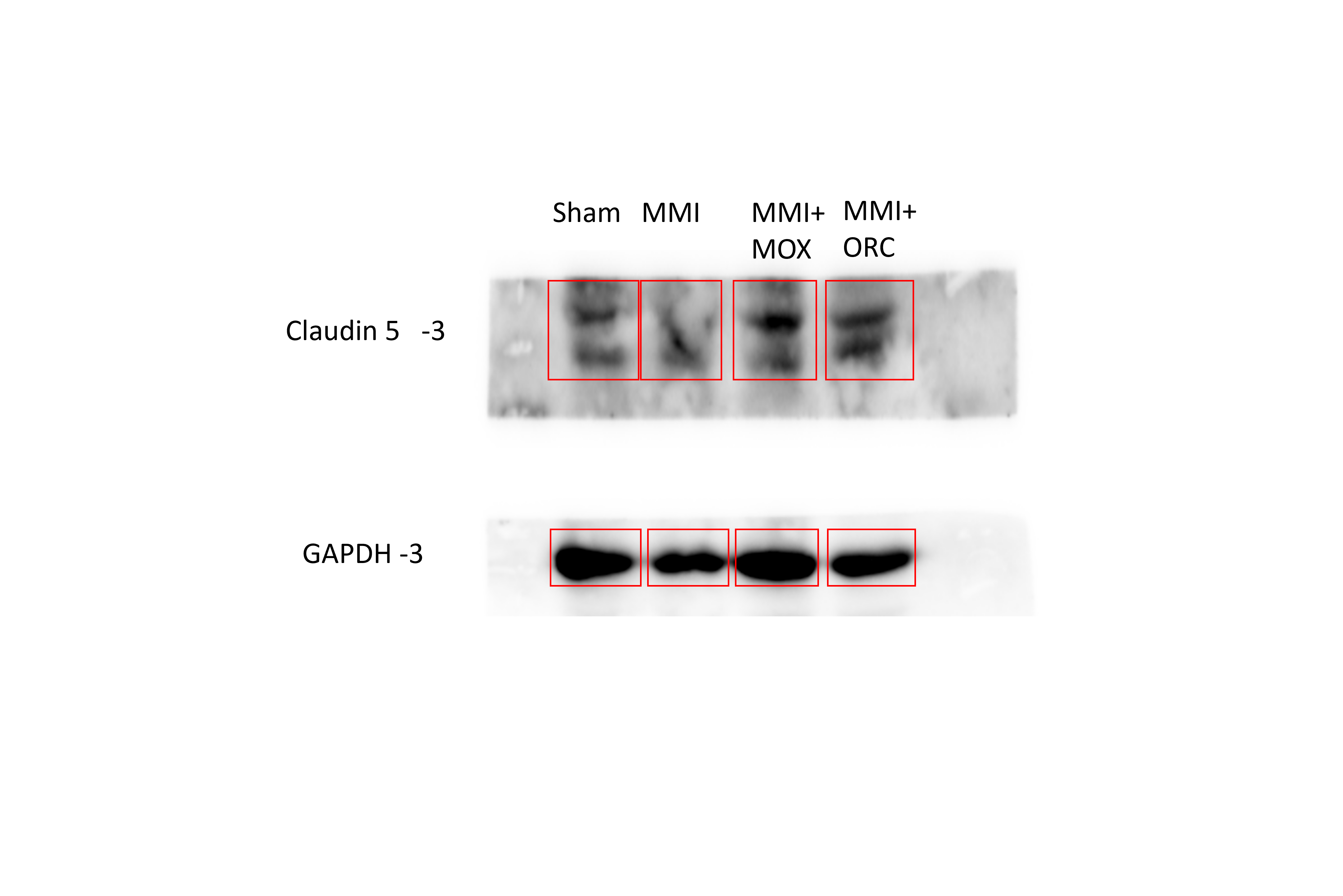

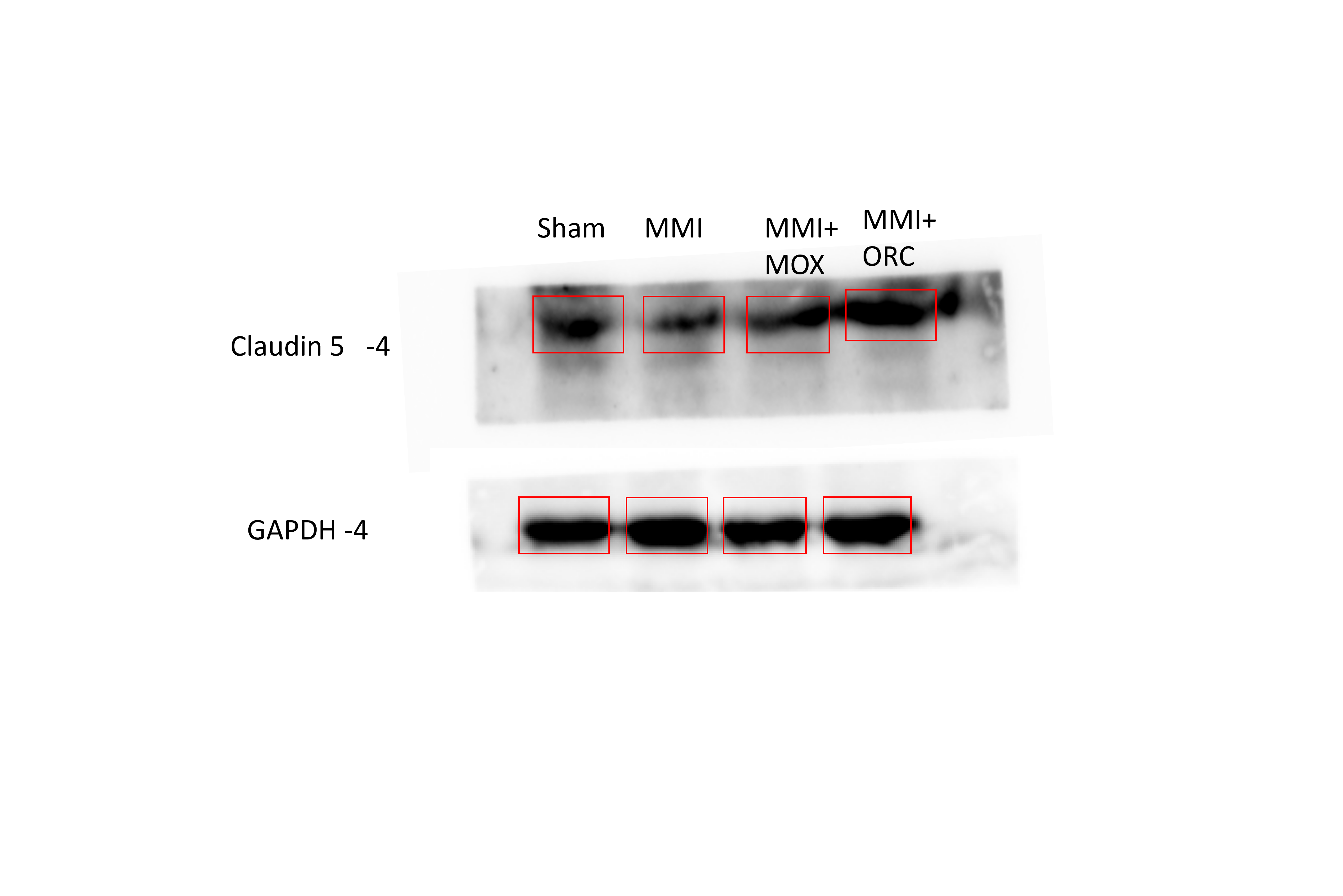


Occludin (Occludin-1 was the representative blot in figures)


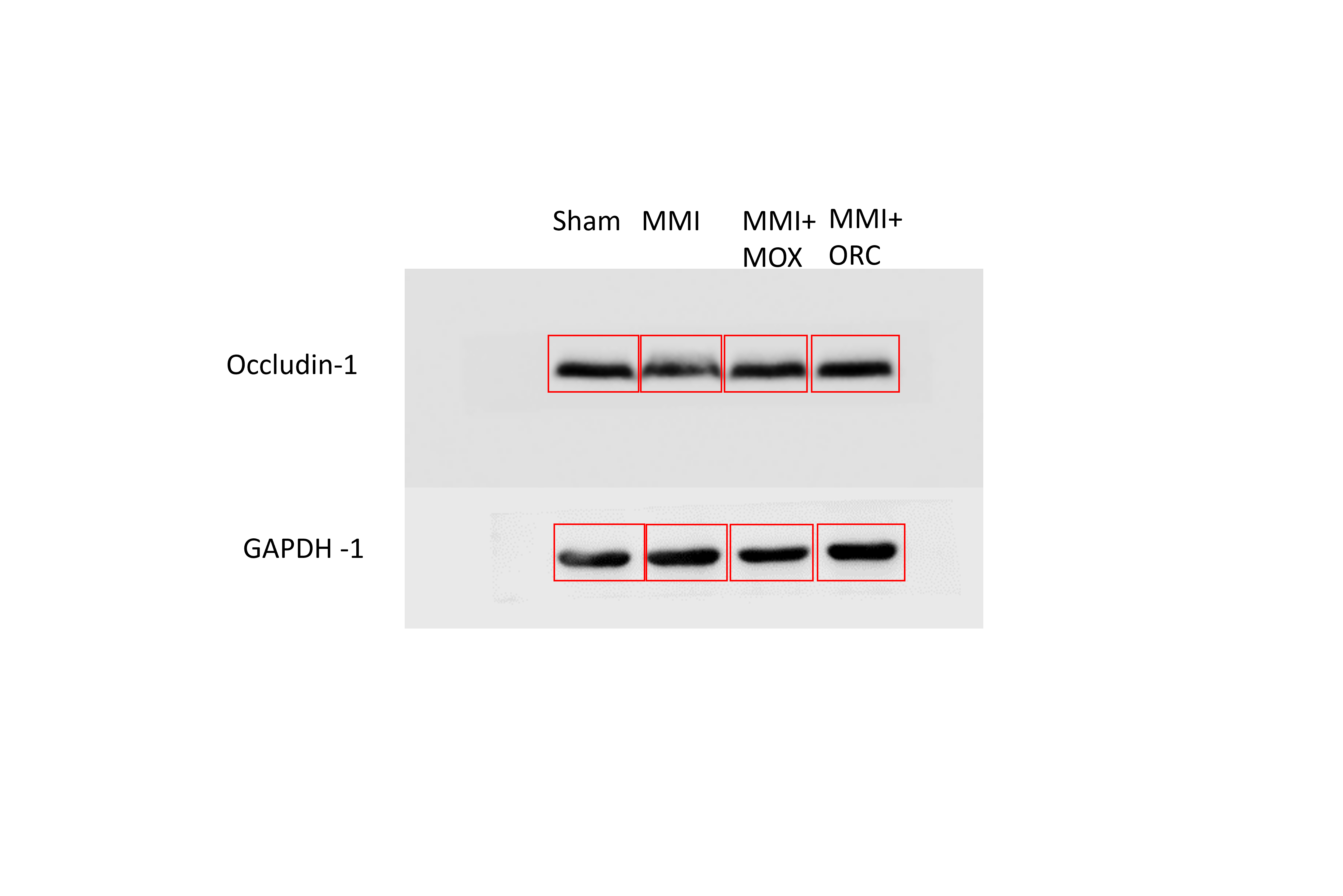

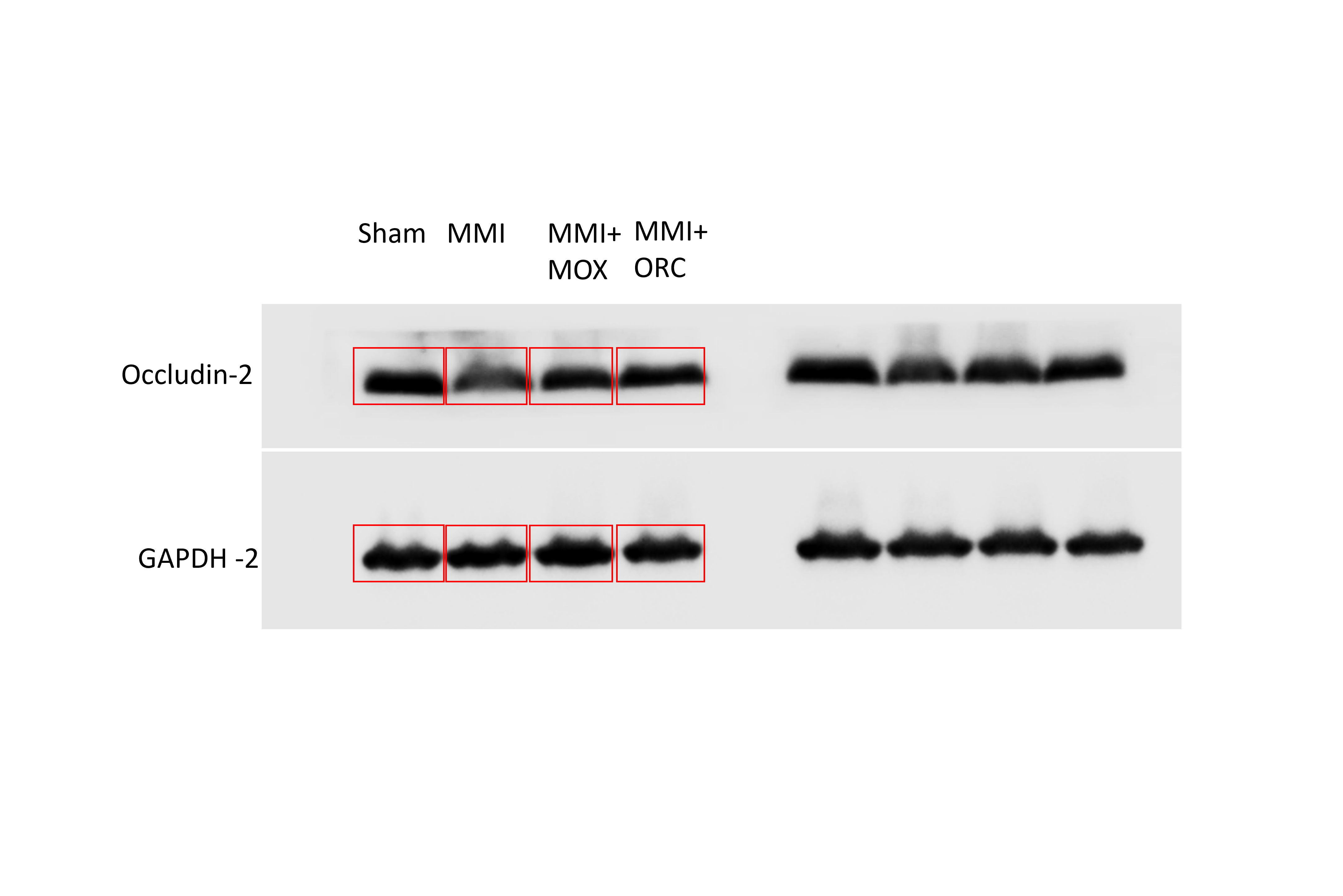


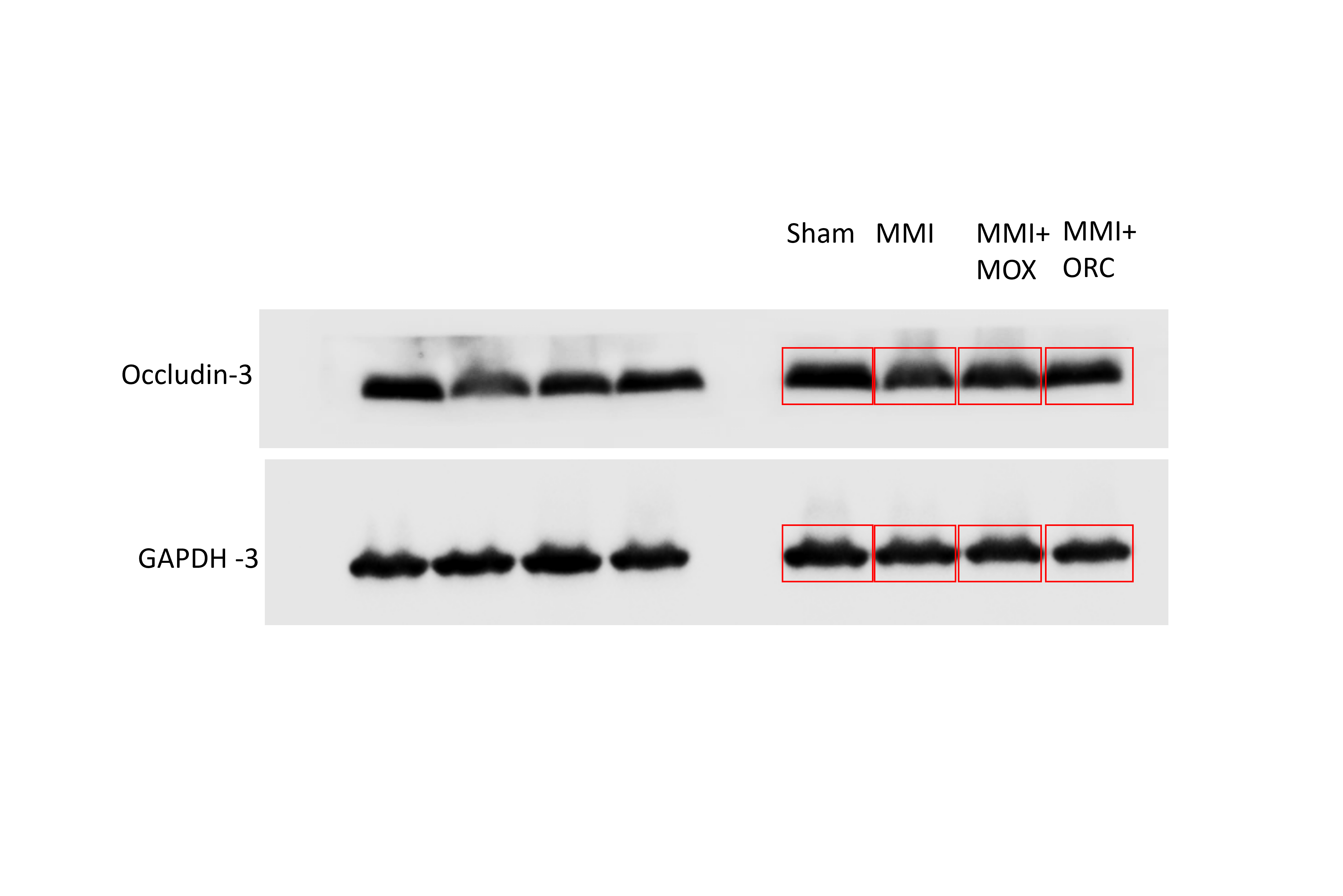

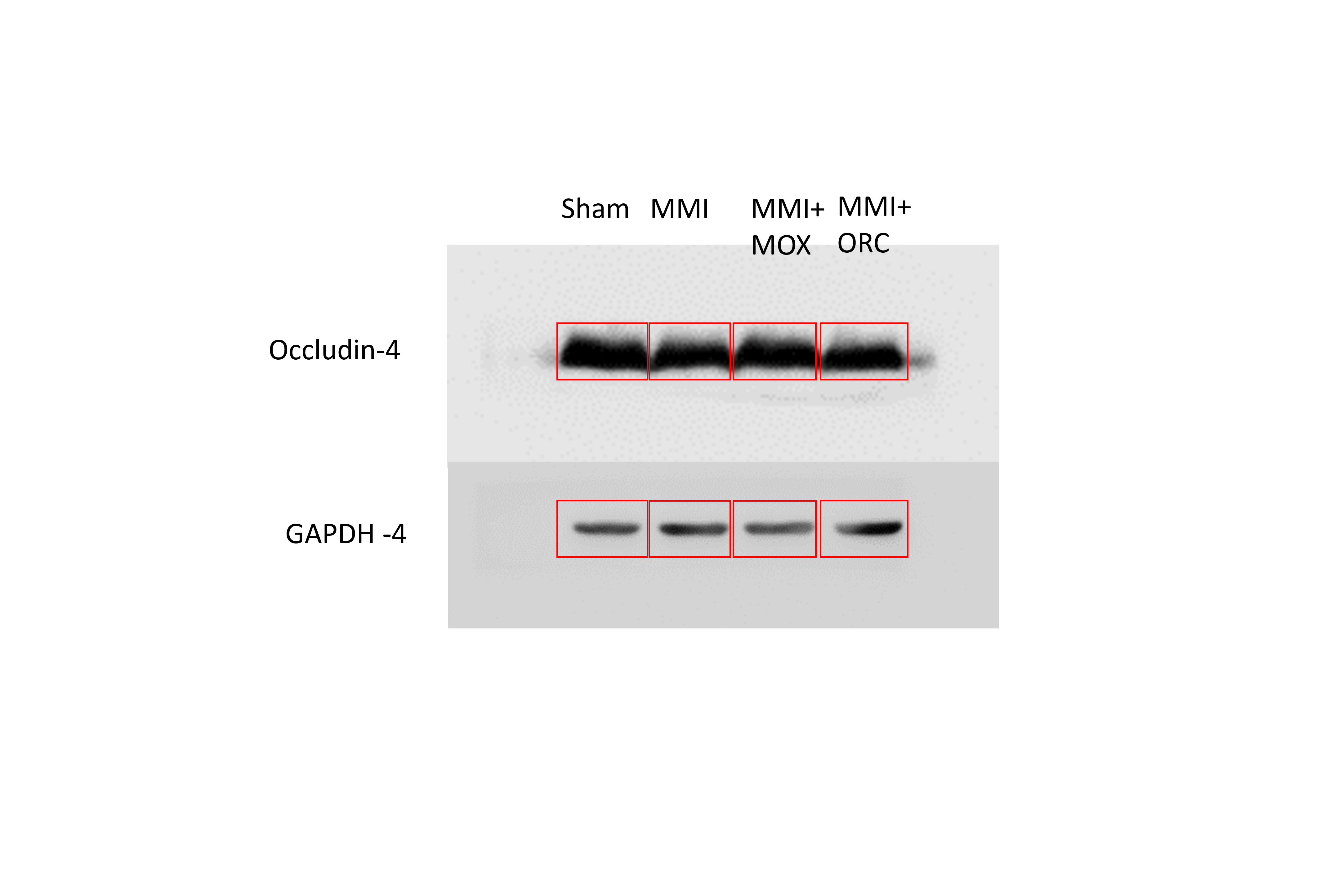

Supplement: Supplementary file 1 [file DataSheet1.DOCX]
